# A Genome-Wide Association Study on the Seedless Phenotype in Banana (*Musa* spp.) Reveals the Potential of a Selected Panel to Detect Candidate Genes in a Vegetatively Propagated Crop

**DOI:** 10.1371/journal.pone.0154448

**Published:** 2016-05-04

**Authors:** Julie Sardos, Mathieu Rouard, Yann Hueber, Alberto Cenci, Katie E. Hyma, Ines van den Houwe, Eva Hribova, Brigitte Courtois, Nicolas Roux

**Affiliations:** 1 Bioversity International, Parc Scientifique Agropolis II, 34397 Montpellier Cedex 5, France; 2 Institute of Biotechnology, Genomic Diversity Facility, Cornell University, Ithaca, NY, 14853, United States of America; 3 Bioversity International, K. U. Leuven, B3001 Leuven, Belgium; 4 Institute of Experimental Botany, Centre of the Region Haná for Biotechnological and Agricultural Research, Olomouc, Czech Republic; 5 CIRAD, UMR AGAP, F-34398 Montpellier, France; Washington University, UNITED STATES

## Abstract

Banana (*Musa* sp.) is a vegetatively propagated, low fertility, potentially hybrid and polyploid crop. These qualities make the breeding and targeted genetic improvement of this crop a difficult and long process. The Genome-Wide Association Study (GWAS) approach is becoming widely used in crop plants and has proven efficient to detecting candidate genes for traits of interest, especially in cereals. GWAS has not been applied yet to a vegetatively propagated crop. However, successful GWAS in banana would considerably help unravel the genomic basis of traits of interest and therefore speed up this crop improvement. We present here a dedicated panel of 105 accessions of banana, freely available upon request, and their corresponding GBS data. A set of 5,544 highly reliable markers revealed high levels of admixture in most accessions, except for a subset of 33 individuals from Papua. A GWAS on the seedless phenotype was then successfully applied to the panel. By applying the Mixed Linear Model corrected for both kinship and structure as implemented in TASSEL, we detected 13 candidate genomic regions in which we found a number of genes potentially linked with the seedless phenotype (i.e. parthenocarpy combined with female sterility). An additional GWAS performed on the unstructured Papuan subset composed of 33 accessions confirmed six of these regions as candidate. Out of both sets of analyses, one strong candidate gene for female sterility, a putative orthologous gene to Histidine Kinase CKI1, was identified. The results presented here confirmed the feasibility and potential of GWAS when applied to small sets of banana accessions, at least for traits underpinned by a few loci. As phenotyping in banana is extremely space and time-consuming, this latest finding is of particular importance in the context of banana improvement.

## Introduction

Banana (*Musa* spp.), including plantain, is a starchy crop cultivated in more than 130 countries. In 2012, the total production of banana in the world reached nearly 140 million tonnes, out of which close to 14% was deemed for export [1]. In addition, banana is the fourth largest food crop in the least-developed countries, and is thus of great importance for food security. Current cultivated bananas arose from a complex domestication scheme that involved several taxa, including different subspecies of *M*. *acuminata* (A genome) and *M*. *balbisiana* (B genome), characterized by high levels of gene flow between these taxa followed by extensive clonal diversification, that is to say the human selection of variants ensuing from the accumulation of mutations or epigenetic changes within clones [2],[3]. In addition, polyploidization also occurred; the initial domestication events resulted in diploids AA or AB, which was followed by the production of triploids AAA, AAB, and ABB through unbalanced meiosis. Most of the popular cultivars such as the commercial AAA Cavendish or the African staple AAB Plantain are triploids. However, quite a number of landraces cultivated in farmers’ fields are diploids.

With the emergence of cost-effective Next-Generation Sequencing technologies and genotyping facilities, an approach like the Genome-Wide Association Studies (GWAS) is becoming widely used in crop plants. Indeed, it has led to the discovery of many candidate genes for traits related to agronomy [4],[5],[6], disease resistance [7], root traits [8] or leaf architecture [9] in cereals. In banana, applying GWAS is challenging due to many deviations from the assumption of Mendelian genetics. Firstly, most of the cultivars are polyploid, for which association studies are challenging due to a lack of appropriate analysis methods, and/or inter-specific hybrids, which complicate SNP mapping and increase genetic heterogeneity. Secondly, cultivated bananas exhibit various rates of fertility, drastically reducing the occurrence of sexual reproduction and therefore limiting sample size. Thirdly, the vegetative mode of propagation and the high rates of clonal diversification of the crop can lead to confounding effects and spurious association due to clonal population structure. These challenges make banana an exciting case study.

**Fig 1:**
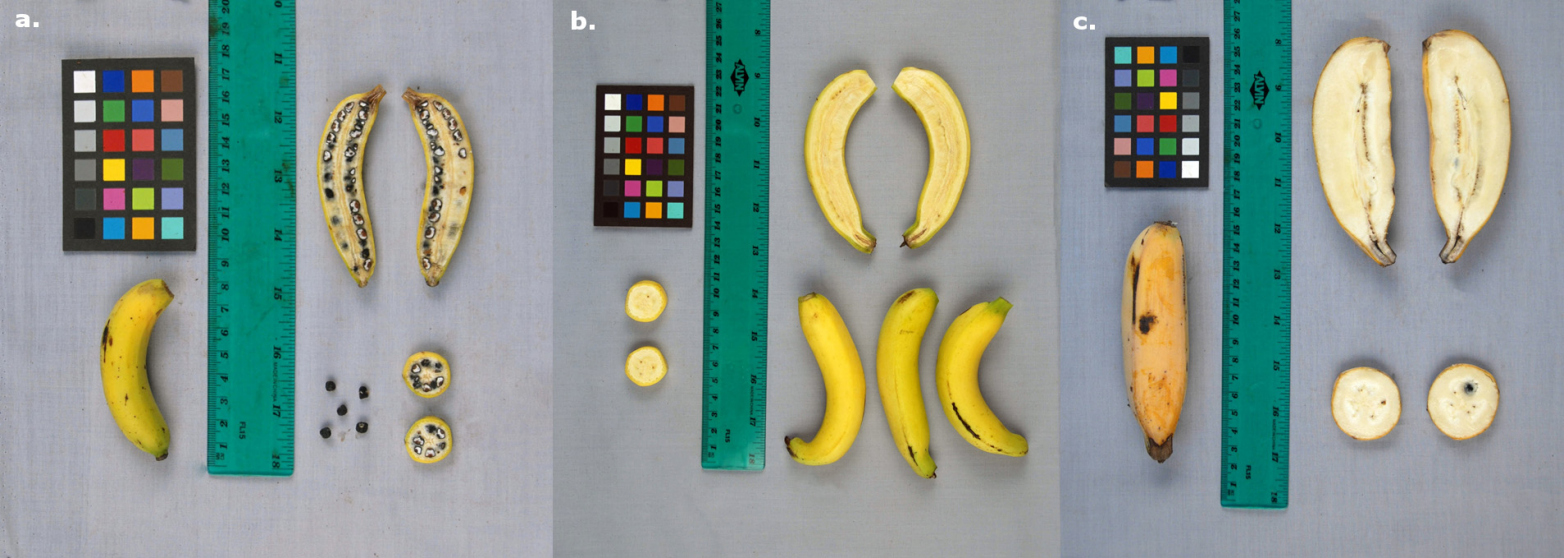
**Fruits of a. *M*. *acuminata banksii* ITC0766 ‘Paliama’, wild and seeded, b. diploid AA ITC1121 ‘Pisang Lilin’ (synonym of ITC0395 ‘Lidi’) cultivated and unseeded and c. triploid ABB ITC0361 ‘Blue Java’ cultivated and occasionally setting seeds.** Credits: B. Irish (USDA-ARS) for the Taxonomic Reference Collection exercise.

While wild ancestors of bananas are mainly out-crossing at the probable exception of *M*. *acuminata banksii* originating in Papua, the main domestication syndrome trait for this crop is the production of seedless and fleshy fruits (Fig 1). This phenotype is due to the ability for parthenocarpy, which is believed to be a contribution of the A genome [10]. This trait corresponds to the capacity of the plant to set and develop fruit without the need of pollination nor subsequent development of seeds that, in non-parthenocarpic plants, are sources of phytohormones required for fruit formation [11],[12]. The plant hormones Auxins (Aux), Gibberellins (GA), Cytokinins and Absicic Acid (ABA) are involved in parthenocarpic fruit development [12],[13],[14]. Parthenocarpy, which can result in the production of seedless fruits, is a desirable trait in many fruit crops and naturally occurs in a number of species. It is driven by different genetic mechanisms according to the species considered, and sometimes even to the lines considered within species. For instance, in summer squash *Cucurbita pepo* and in cucumber *Cucumis sativus*, a single partially dominant gene causes parthenocarpy [15],[16] while in sweet pepper *Capsicum annuum*, a single recessive gene seems to be involved [17]. In eggplant *Solanum melongena*, a major causal gene has also been proposed, but is potentially interacting with a minor one [18]. In tomato *S*. *lycopersicum*, different parthenocarpic lines derive their parthenocarpic status from different genetic sources, some including a single gene [19],[20] and some with a more complex pattern involving several genes [20],[21]. In banana, parthenocarpy is believed to be driven by a major dominant gene (P or P1) interacting with minor ones [22], but there is a clear lack of recent experimental data.

Female sterility is a secondary trait closely linked to banana domestication. It is noticeable that, while all edible bananas are parthenocarpic, they exhibit various levels of female fertility [10],[23] (Fig 1C). The occurrence of total seedlessness can be, in some extreme cases, due to the lack of surrounding pollen for effective pollination rather than to the absence of female fertility. Authors have hypothesized that seedlessness was due to parthenocarpy itself [24],[10], but segregation studies of progenies from different crosses suggested that female sterility in banana was due to a combination of structural and genetic factors, and would be thus an independent and complex character selected by humans concomitantly with parthenocarpy [25], [10].

We report in this paper on a panel composed of 105 genebank accessions that was specifically selected based on available molecular data [26] to support the application of GWAS to banana. This panel is exclusively composed of diploid accessions with pure *M*. *acuminata* genetic backgrounds, both cultivated and wild types, and was genotyped using Genotyping-By-Sequencing (GBS) [27]. After filtering steps to enrich for high-quality genetic markers, we investigated genetic structure, linkage disequilibrium (LD), tested different models for GWAS and explored candidate genes related to domestication traits.

## Materials and Methods

### Selection of the panel

Based on the genotypic data obtained with 498 DArT markers [26], we assessed the number of clonal lineages present within an initial sample composed of 224 diploid accessions with pure *M*. *acuminata* genetic background, all of which are conserved and available for distribution in the Bioversity International Transit Centre (ITC). For this purpose, the GenoType software [28] was used to compute the genetic distances between all pairs of individuals following the Infinite Allele Model (IAM), considering each missing data as one mutation. According to this model, GenoType identified 275 classes of genetic distances within the initial set of diploids. Based on the frequency histogram of the pairwise genetic distances (S1 Fig), we considered Threshold 9 as the end of the first peak of the distribution, which is assumed to correspond to the genetic divergences between pairs of individuals belonging to the same clone. However, and as the distribution of genetic dissimilarities can stretch after the end of the first peak [29], we assumed 11 as an appropriate threshold to delimitate clonal groups and identified 184 different putative clonal lineages. A clonal lineage ID was then assigned accordingly to each of the samples. A dissimilarity matrix based on the Sokal and Michener [30] index was calculated, from which a neighbor-joining (NJ) tree was constructed using DARwin 5.0 [31] (S1 Fig). Combining these data, we selected a set of 105 individuals belonging to different identified clonal lineages to avoid confounding effects due to common clonal ancestry and including genetically distant accessions to increase association power [32]. This set is composed of 77 cultivars, 3 synthetic hybrids and 25 wild accessions and will hereafter be referred to as the panel. Additional elements of taxonomy are provided when relevant, i.e. subspecies and subgroups (Table 1).

**Table 1:** List of the 105 accessions of the panel, their status, origins (PNG: Papua New Guinea, ISEA: Island-South-East Asia, MSEA: Mainland South-East Asia, NA: Not Applicable refers to synthetic hybrids), Clustering as detected by STRUCTURE (Ad.: Admixed accessions) and rates of observed heterozygosity (He).

ITC code	Name	Status	Origin	Classification (species/group-subspecies/subgroup)	Region of Origin	Cluster	He
**ITC0253**	Borneo	wild	Borneo	*M*. *acuminata microcarpa*	ISEA	Ad.	0.19
**ITC0966**	Zebrina (G.F.)	wild	Indonesia	*M*. *acuminata zebrina*	ISEA	Ad.	0.29
**ITC0415**	Pisang Cici Alas	wild	Indonesia	*M*. *acuminata spp*.	ISEA	3	0.08
**ITC0609**	Pahang	wild	Malaysia	*M*. *acuminata malaccensis*	MSEA	1	0.11
**ITC1348**	Pisang Serun 404	wild	Malaysia	*M*. *acuminata malaccensis*	MSEA	1	0.19
**ITC1349**	Pisang Serun 400	wild	Malaysia	*M*. *acuminata malaccensis*	MSEA	1	0.25
**ITC0393**	Truncata	wild	Malaysia	*M*. *acuminata truncata*	MSEA	Ad.	0.08
**ITC0249**	Calcutta 4	wild	Myanmar	*M*. *acuminata burmannicoïdes*	MSEA	1	0.09
**ITC0074**	Malaccensis	wild	Thailand	*M*. *acuminata malaccensis*	MSEA	1	0.18
**ITC0660**	Khae (Phrae)	wild	Thaïland	*M*. *acuminata siamea*	MSEA	1	0.05
**ITC0629**	Selangor 2	wild	Thailand	*M*. *acuminata spp*.	MSEA	1	0.19
**ITC0406**	Pa Musore no.3	wild	Thaïland	*M*. *acuminata spp*.	MSEA	1	0.12
**ITC0408**	Pa Songkhla	wild	Thaïland	*M*. *acuminata spp*.	MSEA	1	0.14
**ITC0668**	Pa (Musore) no.2 x	wild	Thaïland	*M*. *acuminata spp*.	MSEA	1	0.08
**ITC0069**	type 2	wild	unknown	*M*. *acuminata spp*.	MSEA	Ad.	0.33
**ITC0608**	MUSA ACUMINATA	wild	Australia (Queensland)	*M*. *acuminata banksii*	Papua	Ad.	0.27
**ITC0465**	Waigu*	wild	Indonesia (West Papua)	*M*. *acuminata banksii*	Papua	2	0.10
ITC0606	MUSA ACUMINATA*	wild	Indonesia (West Papua)	*M*. *acuminata banksii*	Papua	2	0.05
**ITC0378**	Higa	wild	PNG	*M*. *acuminata banksii*	Papua	Ad.	0.43
**ITC0602**	Hawain 3*	wild	PNG	*M*. *acuminata banksii*	Papua	2	0.30
**ITC0620**	Banksii*	wild	PNG	*M*. *acuminata banksii*	Papua	2	0.11
**ITC0806**	Banksii*	wild	PNG	*M*. *acuminata banksii*	Papua	2	0.04
**ITC0896**	Musa acuminata banksii	wild	PNG	*M*. *acuminata banksii*	Papua	Ad.	0.46
**ITC0897**	Musa acuminata banksii*	wild	PNG	*M*. *acuminata banksii*	Papua	2	0.12
**ITC0530**	A3617/9	wild	Fiji	*M*. *acuminata spp*.	Papua	Ad.	0.31
**ITC0281**	Akondro Mainty	edible	Madagascar	AAcv	East Africa	Ad.	0.40
**ITC1223**	Mshale	edible	Tanzania	AAcv	East Africa	Ad.	0.40
**ITC0063**	Pisang Tongat	edible	Indonesia	AAcv	ISEA	Ad.	0.33
**ITC0090**	Tjau Lagada	edible	Indonesia	AAcv	ISEA	Ad.	0.36
**ITC0611**	Pisang Berlin	edible	Indonesia	AAcv	ISEA	Ad.	0.36
**ITC0679**	Pisang Sapon	edible	Indonesia	AAcv	ISEA	Ad.	0.31
**ITC0685**	Pisang Pipit	edible	Indonesia	AAcv	ISEA	Ad.	0.34
**ITC0689**	Pisang Bangkahulu	edible	Indonesia	AAcv	ISEA	Ad.	0.35
**ITC0712**	AAcv Rose	edible	Indonesia	AAcv	ISEA	Ad.	0.32
**ITC1157**	Pisang Oli	edible	Indonesia	AAcv	ISEA	Ad.	0.31
**ITC0395**	Lidi	edible	Indonesia (Sumatra)	AAcv	ISEA	Ad.	0.28
**ITC0446**	Pu-te La-Bum	edible	Malaysia (Borneo)	AAcv	ISEA	Ad.	0.34
**ITC0447**	Pu-te Wey	edible	Malaysia (Borneo)	AAcv	ISEA	Ad.	0.38
**ITC0480**	Pisang Buntal	edible	Malaysia (Borneo)	AAcv	ISEA	Ad.	0.30
**ITC0299**	Guyod*	edible	Philippines	AAcv	ISEA	2	0.28
**ITC0434**	Racadag	edible	Philippines	AAcv	ISEA	Ad.	0.37
**ITC0974**	Bata Bata	edible	Philippines	AAcv	ISEA	Ad.	0.39
**ITC1150**	Morong Princesa	edible	Philippines	AAcv	ISEA	Ad.	0.32
**ITC1181**	Binaktong	edible	Philippines	AAcv	ISEA	Ad.	0.28
**ITC1229**	Pamoti On	edible	Philippines	AAcv	ISEA	Ad.	0.34
**ITC1231**	Tudlo Tumbaga	edible	Philippines	AAcv	ISEA	Ad.	0.28
**ITC0310**	Morong Princesa	edible	Philippines	AAcv—Pisang Jari Buaya	ISEA	Ad.	0.32
**ITC0316**	Saing Todloh	edible	Philippines	AAcv—Pisang Jari Buaya	ISEA	Ad.	0.40
**ITC0437**	Pisang Kermian	edible	Malaysia	AAcv	MSEA	Ad.	0.32
**ITC0312**	Pisang Jari Buaya	edible	Malaysia	AAcv—Pisang Jari Buaya	MSEA	Ad.	0.32
**ITC0533**	Kluai Lep Mu Nang	edible	Thaïland	AAcv	MSEA	Ad.	0.38
**ITC1358**	Ngu	edible	Vietnam	AAcv—Sucrier	MSEA	Ad.	0.37
**ITC0258**	Pisang Madu	edible	Malaysia	AAcv	MSEA / ISEA	Ad.	0.17
**ITC0568**	Malaysian Blood	edible	Malaysia	AAcv	MSEA / ISEA	Ad.	0.35
**ITC1192**	Diploide EMBRAPA 204	seeded	NA	AA	NA	Ad.	0.43
**ITC1193**	Diploide EMBRAPA 205	seeded	NA	AA	NA	1	0.27
**ITC1267**	IRFA 905	edible	NA	AA	NA	Ad.	0.32
**ITC0292**	Djum Tau	edible	Indonesia (West Papua)	AAcv	Papua	Ad.	0.30
**ITC0318**	Not Named	edible	Indonesia (West Papua)	AAcv—Pisang Jari Buaya	Papua	Ad.	0.27
**ITC0266**	Sowmuk	edible	PNG	AAcv	Papua	Ad.	0.38
**ITC0267**	NBA 14	edible	PNG	AAcv	Papua	Ad.	0.34
**ITC0294**	Pitu	edible	PNG	AAcv	Papua	Ad.	0.49
**ITC0373**	Uwati	edible	PNG	AAcv	Papua	Ad.	0.44
**ITC0589**	Gulum*	edible	PNG	AAcv	Papua	2	0.22
**ITC0600**	Waimara*	edible	PNG	AAcv	Papua	2	0.20
**ITC0603**	Somani*	edible	PNG	AAcv	Papua	2	0.21
**ITC0612**	Mambee Thu	edible	PNG	AAcv	Papua	Ad.	0.43
**ITC0770**	Navaradam*	edible	PNG	AAcv	Papua	2	0.16
**ITC0773**	Mpiajhap*	edible	PNG	AAcv	Papua	2	0.15
**ITC0778**	Gorop*	edible	PNG	AAcv	Papua	2	0.16
**ITC0788**	Katual Vunalir	edible	PNG	AAcv	Papua	na	na
**ITC0797**	Pama*	edible	PNG	AAcv	Papua	2	0.29
**ITC0798**	Garunga*	edible	PNG	AAcv	Papua	2	0.18
**ITC0809**	Maleb*	edible	PNG	AAcv	Papua	2	0.19
**ITC0810**	Sihir*	edible	PNG	AAcv	Papua	2	0.20
**ITC0816**	Kenar*	edible	PNG	AAcv	Papua	2	0.18
**ITC0818**	Enar*	edible	PNG	AAcv	Papua	2	0.14
**ITC0819**	Uyam*	edible	PNG	AAcv	Papua	2	0.15
**ITC0840**	Kuspaka	edible	PNG	AAcv	Papua	Ad.	0.32
**ITC0847**	Hova*	edible	PNG	AAcv	Papua	2	0.19
**ITC0849**	Sepi*	edible	PNG	AAcv	Papua	2	0.18
**ITC0850**	Inori*	edible	PNG	AAcv	Papua	2	0.15
**ITC0869**	Mala	edible	PNG	AAcv	Papua	Ad.	0.31
**ITC0882**	Kwosriake	edible	PNG	AAcv	Papua	Ad.	0.39
**ITC0884**	Awondaeke	edible	PNG	AAcv	Papua	Ad.	0.41
**ITC0886**	Himone	edible	PNG	AAcv	Papua	Ad.	0.33
**ITC0887**	Grupnai	edible	PNG	AAcv	Papua	Ad.	0.38
**ITC0888**	Wikago	edible	PNG	AAcv	Papua	Ad.	0.37
**ITC0889**	Odwa	edible	PNG	AAcv	Papua	Ad.	0.33
**ITC0892**	Paika	edible	PNG	AAcv	Papua	Ad.	0.26
**ITC0893**	Adina	edible	PNG	AAcv	Papua	Ad.	0.32
**ITC0894**	Tainga	edible	PNG	AAcv	Papua	Ad.	0.35
**ITC0923**	Yapu Yapu*	edible	PNG	AAcv	Papua	2	0.17
**ITC0939**	Fu Des	edible	PNG	AAcv	Papua	Ad.	0.35
**ITC0943**	Kwaro	edible	PNG	AAcv	Papua	Ad.	0.37
**ITC0949**	Wiliman*	edible	PNG	AAcv	Papua	2	0.18
**ITC0951**	Te'engi*	edible	PNG	AAcv	Papua	2	0.28
**ITC0984**	Yanun Yefan*	edible	PNG	AAcv	Papua	2	0.16
**ITC1013**	Sena*	edible	PNG	AAcv	Papua	2	0.19
**ITC1015**	Meinje*	edible	PNG	AAcv	Papua	2	0.25
**ITC1187**	Tomolo*	edible	PNG	AAcv	Papua	2	0.19
**ITC1206**	Spiral*	edible	PNG	AAcv	Papua	2	0.15
**ITC1220**	Yalumia*	edible	PNG	AAcv	Papua	2	0.24
**ITC1243**	Kokopo	edible	PNG	AAcv	Papua	Ad.	0.36
**ITC1244**	Mapua*	edible	PNG	AAcv	Papua	2	0.17

* Accessions included in the Papuan subset.

### Phenotype determination

Phenotypic data were based on the known status of each accession with regard to cultivation and wildness (information accessible through MGIS http://www.crop-diversity.org/mgis). In banana, this status is determined based on the presence or absence of seed in the pulp of the developed fruit (Fig 1). When introduced to the ITC genebank, the status of this non-reversible phenotype was provided by germplasm collectors. The phenotypes used in this study were scored as 1 (presence of seeds) or 0 (absence of seeds). In total, the panel is composed of 27 seeded accessions and 78 unseeded accessions. As stated above, such seedless phenotypes can actually be associated with two traits: parthenocarpy, which is fully genetically determined, and female sterility, which seems only partly genetically driven.

### DNA isolation, construction of GBS libraries and high throughput sequencing

Genomic DNA was extracted from lyophilized plant tissues using NucleoSpin 96 Plant II kit (Macherey-Nagel, Duren, Germany), following the manufacturer's instructions. Quality of genomic DNA was checked by agarose gel electrophoresis and quantity was estimated using Nanodrop ND-1000 spectrophotometer (Thermo Scientific, Wilmington, USA).

Two genomic libraries (96 + 9 including one negative control each), were prepared using the Pst1 restriction enzyme according to the procedures published in [27], with the modification that adapters were designed with a PstI overhang (S1 Table). Libraries were sequenced on the Illumina HiSeq2000, version 3 chemistry. Each library was sequenced on two flowcell lanes to achieve the equivalent of 48-plex pooling. All these steps were performed at the Cornell University Biotechnology Resource Center (BRC).

### Read mapping

Raw fastq files were processed with the standalone TASSEL-GBS (version 3.0.160) [33] in preparation for alignment and SNP discovery. Illumina raw reads were assigned to their unique samples using the sample-specific barcodes present in each read. Barcode length ranged between 5 and 10 bases. Only reads that harbored an exact barcode match immediately followed by the expected remnant cut site were kept. Prior to the alignment steps, the reads were trimmed to 64 bases (including the remnant cut site). Reads containing base calls of the indeterminate nucleotide ‘N’ in the first 64 bases were discarded. If reads contained the beginning of the common adapter or a second cut site, they were trimmed to the beginning of the adapter start or second restriction site. Processed reads were then collapsed into a set of unique sequence tags. The minimum number of times a tag must be present across all samples was set to 5, and tags that did not meet this threshold were removed prior to alignment. The number of times each tag was seen for each sample was recorded with an upper limit of 127. Short reads were mapped on the *Musa acuminata* genome assembly reference v1, corresponding to ‘DH Pahang’ the artificial doubled haploid obtained from ITC0609 ‘Pahang’, a wild accession belonging to *M*. *acuminata malaccensis*, [34] available in the Banana Genome Hub [35] (http://banana-genome.cirad.fr/) using BWA v0.7.5a-r405 [36] with default parameters.

### SNP discovery, genotype calling and filtering

SNP discovery was performed with the standalone TASSEL-GBS. Only uniquely aligned reads were considered for SNP calling. Genotype calling was carried out with the TASSEL-GBS plugins ‘tbt2vcfPlugin’ and ‘MergeDuplicateSNP_vcf_Plugin’ with a maximum of 4 alleles per SNP locus.

VCFtools (v0.1.13) and custom Perl scripts were used to filter the 129,658 SNP markers resulting from TASSEL-GBS. Out of the 105 genotypes selected, one exhibited more than 50% of missing data (ITC0788 ‘Katual Vunalir’) and was discarded from the analysis. Given the heterogeneous nature of our species, we decided to set stringent parameters. We thus discarded all markers with a call rate below 100% from the analysis (101,690 SNPs (78.4%) removed) and also excluded markers exhibiting three and four alleles in the whole dataset (5,512 SNPs (4.3%) removed) while one SNP-deletion was considered as a regular allele and recoded as a fictive base to allow the concerned sites to be kept for further analysis (1,001 markers (0.7%) affected). Inbreeding coefficients (F_is_) were calculated for each marker following [37] with the software Genetix 4.05.2 [38]. All markers not following the Gaussian distribution of the F_is_ values (687 SNPs (0.5%) removed). Genotypes with read depth <10 were set to missing (30,419 genotypes across 7,548 SNPs), and markers exhibiting more than nine accessions with missing genotypes were discarded (674 SNPs (0.4%) removed). Finally, we only allowed for a Minor Allele Frequency (MAF) ≥ 0.05 and conserved for further analysis a set of 5,544 highly-reliable markers shared by the panel of 104 genotypes. The rate of heterozygosity per individual was then calculated.

### Analysis of the population structure and kinship coefficient

To save computation time, genotypic data available on 498 DArTs markers were analyzed using the Bayesian Markov Chain Monte Carlo (MCMC) approach coded in the program STRUCTURE version 2.3.4 [39]). The admixture model with the assumption of correlated allele frequencies between groups [40]) was used and 8 replicates of each value of K ranging from 1 to 10 were run with a burn-in-length of 150,000 followed by 1,000,000 iterations of each chain. STRUCTURE then partitioned individuals of the sample according to the membership coefficient Q, that ranges from 0 (lowest affinity to the group) to 1 (highest affinity to the group), across the pre-defined K groups. The most likely true of the values of K was determined by examining the evolution of the posterior probability of the data, named Ln P(D), across the different values of K tested. STRUCTURE was then run following the same model for the best of the values of K identified, that is to say K = 4, using the genotypes obtained with the 5,544 SNPs identified in this study. For the 5 replicates, the burn-in length was of 150,000 followed by 200,000 iterations of each chain.

Pairwise kinship estimates were calculated using the genotypes obtained with the 5,544 SNPs and the simple matching index implemented in DARwin 5.0. The pairwise distance matrix generated was further converted and scaled to a similarity matrix. A dendrogram was also constructed using the weighted NJ algorithm [41] with 1,000 bootstraps.

### Linkage Disequilibrium (LD) calculation and investigation

To evaluate the resolution expected during the GWAS analysis, we investigated the LD pattern through the estimation of the squared allele frequency correlations (r²). We used a two marker Expectation-Maximisation (EM) algorithm, ignoring missing data to estimate the maximum-likelihood values of the four gamete frequencies as implemented in Haploview 4.2 [42], and from which r² and corresponding LOD values were derived from the set of 104 diploid genotypes. Significant values correspond to r² with associated LOD > 2. To better visualize LD decay, significant r² values calculated on the haplotypes and obtained from linked markers, i.e. on the same chromosome, were then plotted against the physical distance (bp) between markers. A generalized additive model (GAM) was applied to our data using ggplot2 module (version 0.9.3.1 http://ggplot2.org/) of the R package to generate smoothened LD curves. A critical value for r² was estimated by square root transforming the unlinked r² values, i.e. between markers separated by more than 10Mb, to obtain a normally distributed random variable. The parametric 95^th^ percentile of that distribution was taken as a critical value of r² value, beyond which LD was likely to be caused by genetic linkage.

### Genome Wide Association Study

The edible or wild status of each accession of the panel was coded as a binary phenotype. To perform GWAS on the full panel, we compared three models for their capacity to fit the data, and tested different correction types for each of these models. Firstly, a simple General Linear Model (GLM) not taking into account the structure of the sample was used. Secondly, we tested a Mixed Linear Model (MLM) using the kinship matrix (K) as the random effect (MLM K). Thirdly, we tested a MLM taking into account both K and Q (MLM K + Q) using the Q matrix obtained with STRUCTURE for the 5544 SNPs for K = 4. Finally, to minimize false positive and negative associations due to low allele frequencies, each of these models was run with two levels of filtering on MAF 5% and 10% corresponding to 5,544 and 3,912 SNPs. To determine which of the models and correction parameters tested were best fitting the data, we plotted the negative log10-transformed observed p-values obtained for each SNP association against their expected distribution under the null hypothesis of no genetic association (Quantile-Quantile plot). Manhattan plots were displayed accordingly. We then considered both nominal and adjusted p-values. Adjustment was performed by using Bonferroni-corrected critical p-values (5% and 1%). To moderate the stringency of the Bonferroni adjustment, q-values, corresponding to the False Discovery Rate (FDR), were computed using the SFDR software [43]. In order to validate the results obtained while analyzing the subset, we ran a second GWAS using a second, unstructured dataset using a simple GLM and a MLM K. This dataset was composed of a subset of 33 individuals including 27 cultivated and 6 wild accessions that were identified by STRUCTURE as the largest group of unadmixed (Q>0.8) accessions. It will hereafter be referred to as the subset. Interestingly, all the 33 individuals of the subset but one originates from Papua. This analysis was run with polymorphic SNPs with MAF 5% and 10% (2518 and 2039 SNPs respectively). Due to the small size of the sample, we flagged it as positively associated with the studied phenotype those of the SNPs that passed the Bonferroni correction only.

### Identification of Candidate genes

The positions of the markers significantly associated to the seedless phenotype were used as starting points to explore the genomic context of the *M*. *acuminata* reference sequence. With regard to the pattern of LD decay, we scanned the genome on a window of about 40 kb upstream and downstream the SNPs of interest to identify the genes located in the regions. In the case where two closely distant SNPs showed association (i.e. on chromosome 4 and on chromosome 7), the entire genome region located between these SNPs was explored. All the gene annotations in the selected regions were checked for their structural annotation and exon-intron boundaries were modified whenever necessary. In some cases, genes missed by the automatic annotation were added. Next, to verify the accuracy of the functional annotation assignment of the *Musa* genes, we identified homologous genes in other plant species, mainly *Oryza sativa*, *Vitis vinifera* and *Arabidopsis thaliana* using the GreenPhyl comparative genomics database [44] (http://www.greenphyl.org) or with a BLASTP as described in [45]. Ortholog gene identification was fine-tuned using a phylogenomic pipeline performed using a South Green Galaxy workflow available at http://galaxy.southgreen.fr/galaxy/u/delphine-l/w/greenphyl-phylogenomic-analysis-workflow-imported-from-uploaded-file. It included a multiple alignment (MAFFT v.6 [46]) followed by a masking step (GBLOCKS [47]). Then, gene tree reconstruction was performed using PhyML [48] with tree improvement with best of NNI methods with aLRT support [49].

## Results

### Marker distribution

The GBS method and the filtering pipeline applied to the dataset yielded 5,544 markers (4,543 SNPs and 1,001 one base-indels) with an average nucleotide diversity (Pi) of 0.29. Even though markers with a MAF below 5% were discarded, the MAF distribution of the markers was skewed toward low frequencies with 31.1% of the markers displaying a MAF below 10%. Approximately 69% of these markers were located into genic regions (i.e. 5’ UTR, CDS, intron or 3’ UTR). The heterozygosity rates for each individual of the panel ranged between 0.04 and 0.53 (Table 1). We observed an average rate of 0.19 (from 0.04 in ITC0806 ‘Banksii’ to 0.46 in ITC0896 ‘Musa acuminata banksii’) in the wild-types and of 0.29 (0.14 for ITC0818 ‘Enar’ to 0.49 for ITC0294 ‘Pitu’) for the edible types. The average density of markers corresponded to an average of one marker each 60 kb, with a minimum of one marker every 49 kb on chromosome 1 and a maximum of one marker every 71 kb on chromosome 10 (S2 Fig). We noted that 65.8% of the intervals were less than 20 kb and 83.3% were less than 100 kb. However, 94 segments greater than 500 kb without any markers were identified, including one of more than 3 Mb on chromosome 9.

### Panel structure

**Fig 2:**
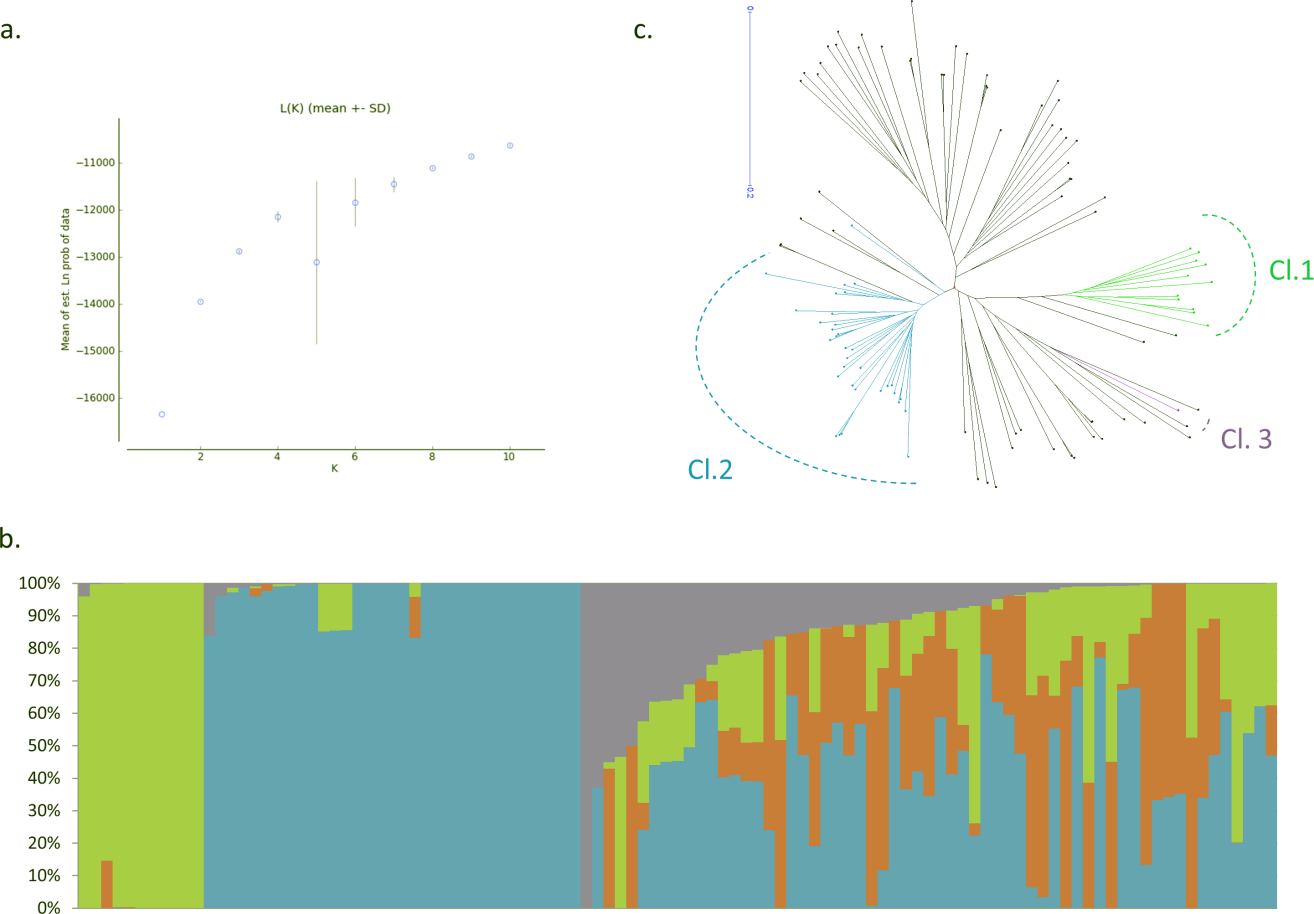
Genetic structure of the panel. STRUCTURE outputs: a. LnP(D) and its variance across 8 runs for each values of K, from 1 to 10, based on the genotypic data available for 498 DArT markers and b. Representation of the admixture pattern of the panel for K = 4 as calculated on the genotypic data obtained with the 5544 SNPs of the study, each bar corresponds to a genotype and colours correspond to the detected genetic clusters. c. NJ tree conducted on a Simple Matching dissimilarity matrix. Coloured branches correspond to unadmixed accessions (Q > 0.8) and black branches to admixed accessions (Q< 0.8) at K = 4.

The genetic structure of the panel was investigated following the model based approach implemented in STRUCTURE. Firstly, we used a dataset of 498 DArT markers already available to determine the most likely true of the numbers of genetic clusters (K) present in the panel. The LnP(D) appeared to be a stable increasing function of K for all the values observed from 1 to 4 and started to exhibit high variance at K = 5 and then stabilize newly at K = 8. We therefore considered K = 4 as the most likely true of the values of K tested. At K = 4, the first cluster is composed of 11 unadmixed (Q>0.8) accessions including 10 wild types originating in SE-Asia and one improved material ITC1193 Diploid EMBRAPA bearing seeded fruits. The cluster 2 is the widest and is composed of 33 unadmixed accessions including 27 cultivated originating in Papua at the exception of one, ITC0299 Guyod originating in the Philippines, and 6 wild accessions belonging to the subspecies *banksii*. The cluster 3 is composed of one unadmixed accession only, ITC0415 ‘Pisang Cici Alas’, which is a wild specimen belonging to the species *M*. *acuminata* but not affiliated to any subspecies. None of the accessions belong to the fourth group, which only exists, in the panel, under the form of introgressions within admixed accessions. Noticeably, 59 of the 104 accessions composing the panel appeared admixed between 2 or more clusters, revealing sometimes complex admixture patterns (Fig 2B). Out of these 59 admixed accessions, 8 were wild types. Interestingly, all the cultivated accessions originating in SE-Asia except ITC0299 Guyod were admixed between several genepools (Table 1). Therefore, the set of 33 unadmixed accessions mostly originating from Papua was considered for subsequent analyses as a subset of the panel and was used to assess the impact of the complex genetic structuring of the panel on the association study. The genetic distances calculated from the 5,544 SNPs then allowed the construction of a NJ tree on which we projected the clustering obtained with STRUCTURE for K = 4 (Fig 2C).

We calculated the mean He for the clusters identified by STRUCURE. Cluster 1, exclusively composed of seeded accessions, exhibited an average He of 0.15, while Cluster 2, the Papuan subset, has an average He of 0.18 (0.12 for wild types and 0.19 for edible types). The only accessions of Cluster 3 has a He of 0.08 and the admixed accessions had overall an average He of 0.34 (0.29 for the wild types and 0.35 for the edible).

### Pattern of LD

**Fig 3:**
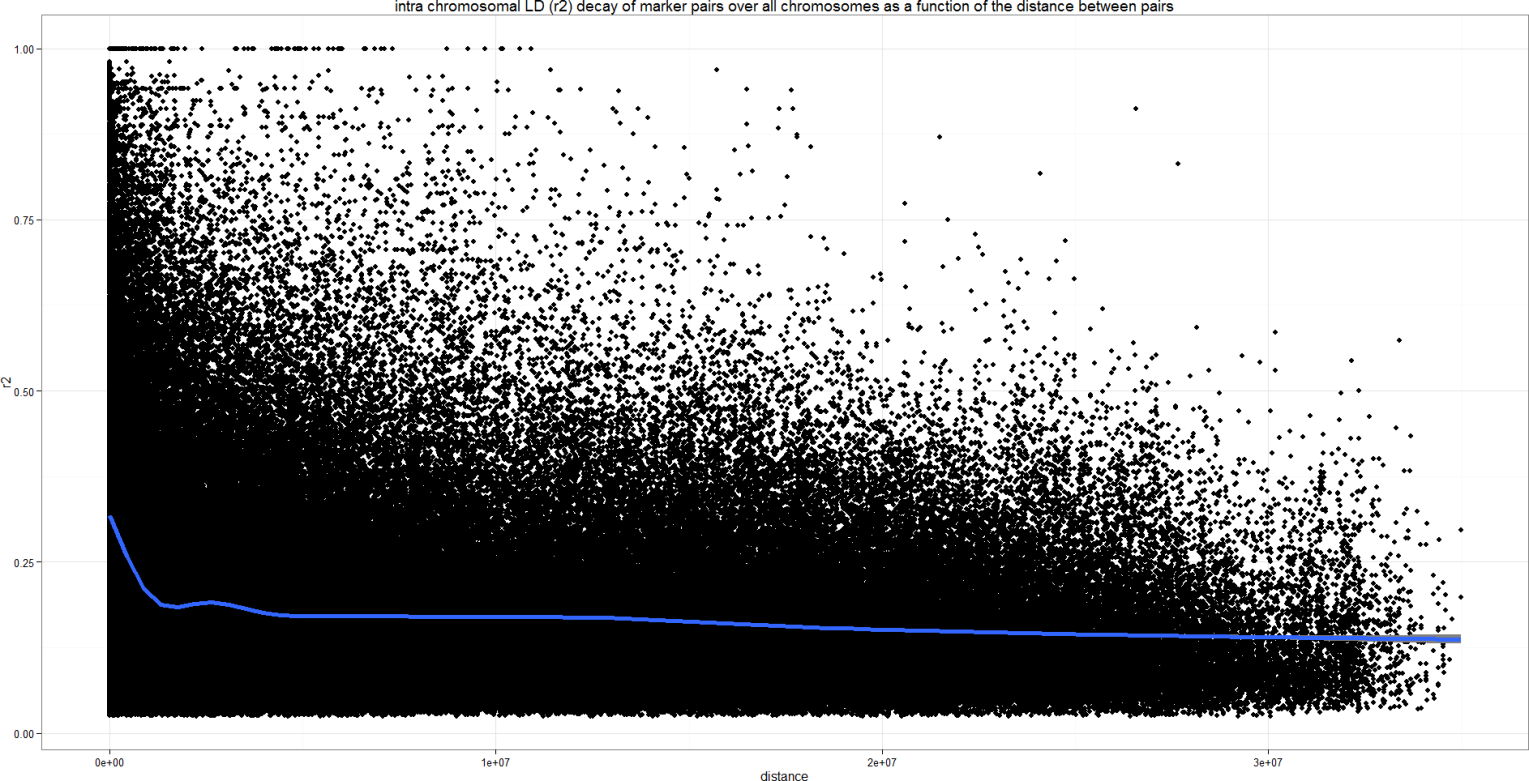
Scatter plots of LD (r²) for all 1,417,890 marker pairs as a function of physical distance (bp) for the full panel composed of 104 diploid *Musa* accessions with a pure *M*. *acuminata* genetic background as calculated by HAPLOVIEW. LD decay curve fitted by the GAM function is represented by the blue line.

The analysis performed by the Haploview software on the 104 genotypes, showed that 26.09% of the 1,417,890 marker pairs were in significant LD (LOD>2) In the first distance class corresponding to the 0 to 10 kb interval, the average LDs, expressed as the average r² values, were 0.25 (with 54.03% significant values exhibiting an average r² of 0.47). We noticed that this initial value of r² dropped to half its initial value within 10 to 100 kb and then decreased slowly without reaching background LD, i.e. average r² obtained for markers distant of more than 10 Mb. We noted that 39.35% of the markers pairs are still significantly in LD after 100 kb. The plots of the r² values against the physical distance are presented in Fig 3. The smoothened LD curves, obtained by applying generalised additive models (GAM) to our data, show a secondary increase in r²). These secondary increases in r² values may reflect the occurrence of long-range LD within the panel.

Considering as unlinked the loci separated by more than 10 MB, we attempted to evaluate a critical value of r² above which detected LD cannot be attributed to the structure of the panel only. The 95% percentile threshold based on the r² values obtained from the unlinked markers was set at 0.212. This threshold value is high and reflects high levels of background structure within the panel. As indicators, only 9.14% of the significant r² values stood above this threshold (Table 2).

**Table 2:** LD expressed as squared allele frequency correlation (^r²^) calculated for the panel of 104 banana accessions selected for GWAS and genotyped with 5544 markers.

		Total pairs	Mean r^²^ (total)	%significant pairs	significant pairs	Mean r^²^ (significant pairs)	% of pairs > thr.	Mean of r^²^ > thr.
**Genotypes**	*Total linked*	1417890	0.06	26.09	370032	0.17	9.14	0.56
	0kb-10kb	11001	0.25	54.03	5955	0.47	47.98	0.77
	10kb-100kb	10384	0.12	41.65	4046	0.29	23.43	0.64
	100kb-500kb	44253	0.10	39.35	16394	0.26	19.39	0.59
	500kb-1mb	53351	0.09	34.47	18519	0.24	16.39	0.58
	1mb-5mb	366111	0.07	28.06	105122	0.20	10.50	0.55
	5mb-10mb	297984	0.06	24.74	73975	0.18	7.39	0.53
	10mb-100mb	634806	0.05	22.90	146021	0.17	4.99	0.49

r² values with associated LOD values > 2 are considered significant. Threshold values correspond to the 95^th^ percentile of the distribution of the square root transformation of r² calculated for pairs of markers considered unlinked, i.e. separated by more than 10 Mb.

### Genome-wide association study

**Fig 4:**
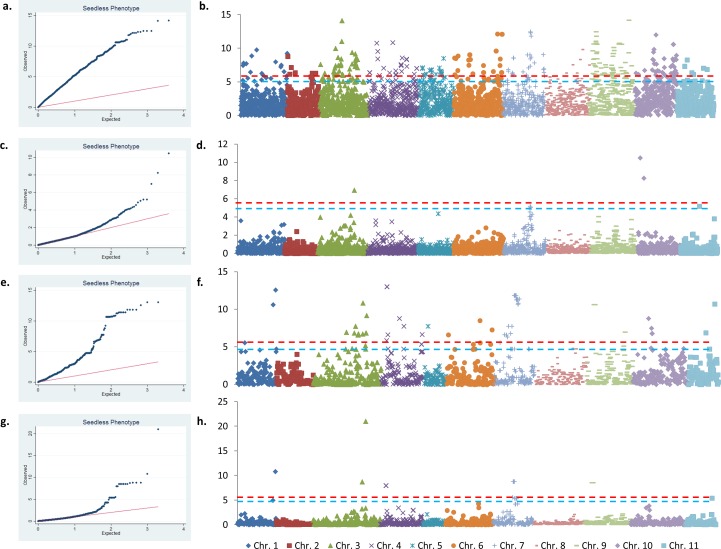
GWAS for the seedless phenotype (MAF > 10%). **Quantile-Quantile plots and corresponding Manhattan plots obtained on the Panel using simple GLM (a-b) and uncompressed MLM K + Q (k = 4) (c-d) and the Papuan Subset using simple GLM (e-h) and uncompressed MLM K (g-h).** Colored dashed lines correspond to Bonferroni corrected thresholds of p = 0.05 (blue) and p = 0.01 (red).

The occasional inter-specific hybridization, the polyploidy of the most popular cultivars and the high rates of clonal diversification due to its vegetative mode of propagation complicate GWAS in banana. To evaluate the potential of a panel composed of 104 diploid and pure *M*. *acuminata* accessions selected for performing GWAS, we carried out an association analysis with the seedless phenotype. To avoid false-association, we performed all subsequent analyses with sets of markers with MAF > 5% (5,544 SNPs for the panel and 2,518 SNPS for the subset) and MAF > 10% (3,912 SNPs for the panel and 2,039 SNPs for the subset). Different statistical models and correction parameters for each model were tested on the panel and the subset, to control spurious LD caused by population structure and to calculate the p-values of marker-trait associations. For both studies, MLM greatly reduced false-positives compared to the GLM as shown by the QQ-plots (Fig 4, S3 Fig). Quantile-Quantile plots and corresponding Manhattan plots obtained when running the association with MAF > 5% are presented in S3 Fig Considering the associations performed on the full panel, the SNPs statistically associated with the seedless phenotype following MLM K + Q (K = 4) are presented in Table 3 along with the ranges of the p-values and q-values they obtained. In total, the GWAS performed on the full panel identified 21 markers, corresponding to 13 genomic regions located on chromosomes 1, 3, 4, 5, 7, 9, 10 and 11, for which associated q-values were below 0.05. Out of these 21 SNPs, 1 located on chromosome 3 and 2 located on chromosome 10 exhibited significant Bonferroni-corrected associations at 1% and 2 SNPs on the same region of chromosome 7 along with one SNP of chromosome 11 were significantly associated at 5% (Table 3). The GWAS performed on the subset identified 23 markers (Table 4), corresponding to 8 genomic regions located on chromosomes 1, 3, 4, 7, 9 and 11, for which p-values were significantly associated after Bonferroni correction at 5%. Out of these 8 genomic regions, 6 were also considered significantly associated with the seedless phenotype while analyzing the full panel. These regions are located on chromosomes 3, 4, 7, 9 and 11. Noticeably, a second SNP of chromosome 4, located at circa 150 kb of the SNP detected during the analysis of the panel, was detected while analyzing the subset. It is noticeable that the proportions of explained phenotypic variance (R²) were quite high for both analysis and in particular when analyzing the subset.

**Table 3:** SNPs statistically associated to the seedless phenotype when analysing the panel with the MLM K + Q (K = 4) and the corresponding ranges of p- and q-values obtained. SNPs in bold are those commonly detected when analyzing the subset. R² is the proportion of phenotypic variance explained by the markers.

SNP ID	Chr	Position	Alleles	Location	Gene name	MAF (%)	p-values	q-values	R²	Effect	Allele
S1_2170475	1	2170475	G/T	Exon	GSMUA_Achr1T02650	10.58	-	+	0.14	-1.24	K
										-1.15	G
S3_971104	3	971104	A/G	UTR	GSMUA_Achr3T01360	50.00	-	+	0.16	0.48	A
										0.01	R
S3_22358632	3	22358632	A/T	Intergenic		38.46	-	+	0.16	0.41	A
										0.08	W
S3_23751148	3	23751148	A/G	Intron	GSMUA_Achr3P22900	10.48	***	++	0.25	-0.74	A
										-0.20	R
S4_5366421	4	5366421	T/C	Exon	GSMUA_Achr4T07200	28.10	-	+	0.14	0.42	C
										0.06	Y
S5_11617038	5	11617038	G/-	Intron	GSMUA_Achr5T15610	18.57	-	+	0.17	0.42	C
										-0.12	G
S7_21735706	7	21735706	G/T	Intron	GSMUA_Achr7T18900	48.57	-	+	0.15	0.50	G
										0.17	K
S7_21735724	7	21735724	-/T	Intron	GSMUA_Achr7T18900	34.29	-	+	0.17	0.45	T
										0.17	A
S7_21735725	7	21735725	T/-	Intron	GSMUA_Achr7G18900	17.14	-	++	0.18	0.49	A
										-0.08	T
S7_21735729	7	21735729	T/C	Intron	GSMUA_Achr7G18900	18.57	**	++	0.19	0.47	C
										-0.10	T
S7_21735767	7	21735767	G/A	Intron	GSMUA_Achr7G18900	18.09	**	++	0.19	0.48	A
										-0.07	G
S7_21742629	7	21742629	C/T	Intergenic		26.44	-	+	0.14	0.40	T
										-0.07	G
S7_21742664	7	21742664	T/G	Intergenic		39.90	-	+	0.15	0.51	T
										0.12	K
S7_21742673	7	21742673	C/G	Intergenic		39.90	-	+	0.15	0.51	C
										0.12	K
S7_21920043	7	21920043	G/A	Intron	GSMUA_Achr7T19120	42.86	-	+	0.16	0.45	G
										-0.02	C
S9_3878154	9	3878154	G/A	Exon	GSMUA_Achr9G06060	11.43	-	+	0.15	-0.39	G
										-0.16	W
S9_30886263	9	30886263	G/A	Exon	GSMUA_Achr9T26460	44.76	-	+	0.14	0.39	G
										0.03	R
S10_5800489	10	5800489	C/T	intergenic		10.19	***	++	0.36	-0.94	Y
										-0.08	C
S10_9284262	10	9284262	C/T	Intergenic		40.59	***	++	0.30	0.80	T
										-0.01	Y
S11_15250304	11	15250304	T/-	Intron	GSMUA_Achr11T14210	42.38	**	++	0.20	-0.70	T
										-0.05	W
S11_24122623	11	24122623	A/G	intergenic		27.14	-	+	0.14	-0.68	A
										-0.59	R
Total number of significant SNPs	15	21									

^¶^: Major/Minor

-: Not applicable

For 3912 markers with MAF>10%, the Bonferroni-corrected thresholds for the p-values were 2.6e-6 (α = 0.01) coded as *** and 1.3e-5 (α = 0.05) coded as ** FDR results (q-values) were coded as ++ (α = 0.01) and + (α = 0.05).

**Table 4:** SNPs statistically associated to the seedless phenotype when analyzing the subset with the MLM K and corresponding range of p-values obtained. SNPs in bold are those commonly detected when anlyzing the panel. R² is the proportion of phenotypic variance explained by the markers.

SNP	Chr.	Position	Alleles	MAF	p-values	R²
S1_18584608	1	18584608	G/A	12.12%	**	0.60
S1_25846792	1	25846792	G/T	21.21%	***	0.69
**S3_22358632**	3	22358632	T/A	36.36%	***	0.67
**S3_23751148**	3	23751148	A/G	13.63%	***	1.00
**S4_5366421**	4	5366421	C/T	19.70%	***	0.86
S4_5515849	4	5515849	-/T	19.70%	***	0.86
**S7_21735706**	7	21735706	T/G	22.73%	***	0.84
**S7_21735725**	7	21735725	T/-	22.73%	***	0.66
**S7_21735729**	7	21735729	T/C	22.73%	***	0.66
**S7_21735767**	7	21735767	G/A	22.73%	***	0.66
**S7_21742629**	7	21742629	C/T	27.27%	**	0.82
S7_21795942	7	21795942	A/G	27.27%	**	0.82
S7_21796003	7	21796003	G/-	50.00%	**	0.77
S7_21796004	7	21796004	A/-	50.00%	**	0.77
S7_21796005	7	21796005	-/T	39.39%	**	0.76
S7_21796006	7	21796006	-/C	39.39%	**	0.76
S9_3878110	9	3878110	A/G	10.61%	***	0.66
S9_3878150	9	3878150	G/T	10.61%	***	0.66
S9_3878151	9	3878151	A/C	10.61%	***	0.66
**S9_3878154**	9	3878154	G/A	10.61%	***	0.66
S9_3878161	9	3878161	A/G	10.61%	***	0.66
S9_3878238	9	3878238	T/A	10.61%	***	0.66
**S11_24122623**	11	24122623	A/G	45.45%	**	0.70

For 2039 SNPs markers with MAF>10%, the Bonferroni-corrected thresholds for the p-values were 4.90e-6 (α = 0.01) coded as *** and 2.45e-5 (α = 0.05) coded as **.

### Identification of candidate genes

**Fig 5:**
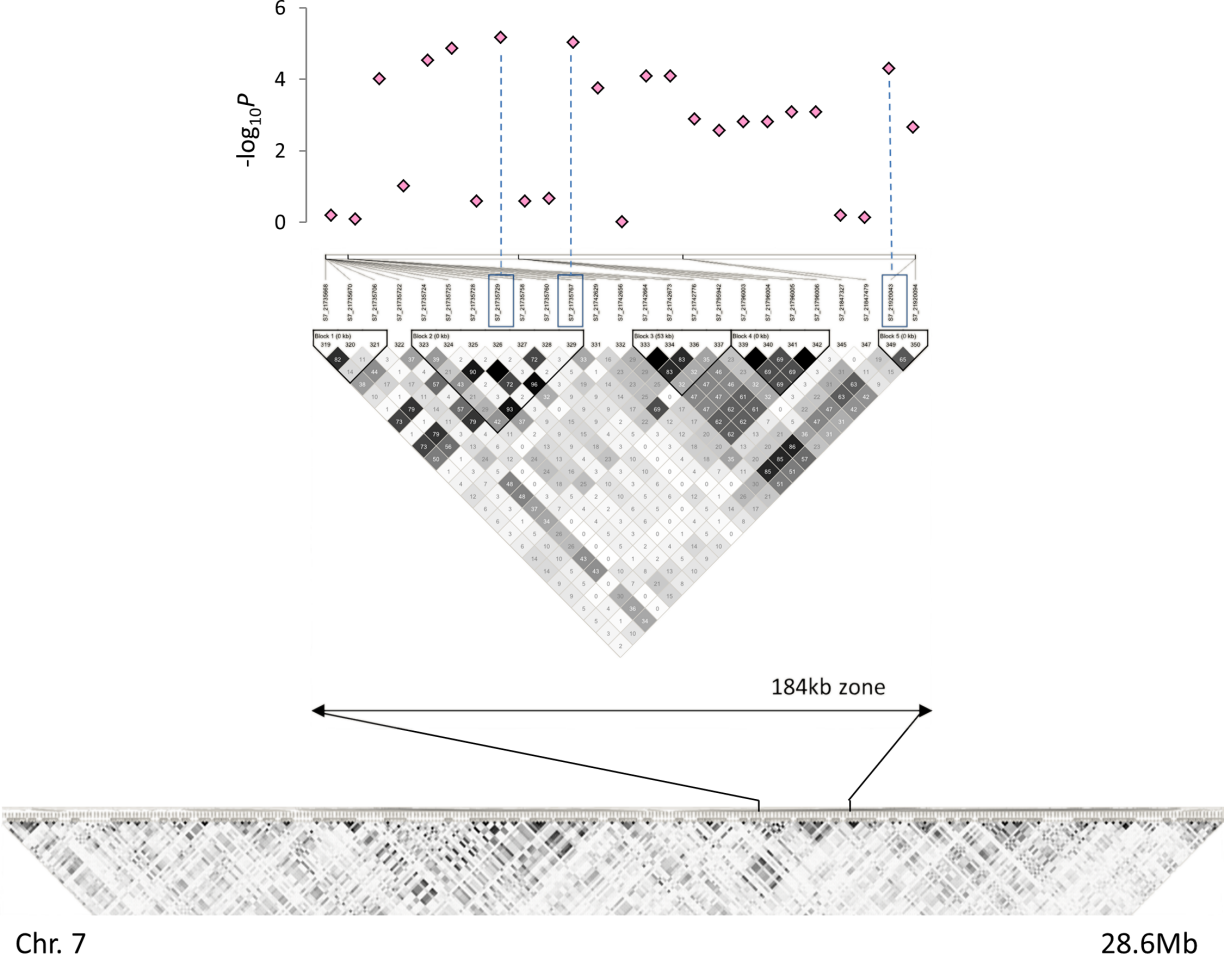
Local association plot for the approximate 184 kb candidate region detected on chromosome 7 and LD maps for the entire chromosome and the corresponding region. Dashed lines indicate the most significant associations.

Taking into consideration that a substantial amount of markers pairs are still significantly in LD after 100 kb and that the markers used in this study are found every 60 kb on average, we scanned for genes of interest the regions surrounding the SNPs significantly associated over 80 kb. In addition, and when specific patterns of association were detected through the GWAS, we explored the full regions between two associated SNPs that were moderately distant, as was the case on chromosome 7, for which we explored the full 184 kb of the region positively associated with the traits (Fig 5). Precise locations of the 21 SNPs detected are presented in Table 3. We noted that 14 were located within genes, while 7 were located into intergenic regions. The analysis of the subset confirmed the regions of chromosomes 3, 4, 7, 9 and 11 as candidates and spotted 2 additional regions on chromosome 1 (Table 4). In addition, on chromosome 4, an additional SNP, corresponding to a deletion and located in an intergenic region located almost 150 kb downstream of the SNPs significantly associated with the panel, was detected. As the two markers on chromosome 4 were found to be consecutive and in full LD in the subset, the 150 kb region was explored. The genome scans of the regions detected through the analysis of the panel, coupled with manual annotations when necessary, yielded a total of 161 genes. The two additional regions of chromosome 1 detected when analyzing the subset were also explored and yielded 15 genes. Detailed results are presented in S2 Table. Gene density in such window can be variable. At the extremes, both regions of chromosome 10 yielded 1 gene only while the region of chromosome 7, the biggest, yielded 33 genes.

Based on comparative genomic approaches and functional annotation coming from experimental evidence, we scanned the genome for genes possibly involved in the hormonal pathway inducing parthenocarpy or orthologous to genes that were demonstrated to be associated to flower and fruit development or fertility in plants. Interesting candidates related to our phenotypes were identified on chromosomes 1, 3, 4, 5 and 7 but none on chromosome 9, 10 and 11 (Table 5). Out of these 11 genes, one is potentially linked to Auxin signaling (GSMUA_Achr4P07220_001 [50]), two to Gibberelin signaling (GSMUA_Achr4P07190_001 and GSMUA_Achr4P07320_001) and two to Abscisic Acid signaling (GSMUA_Achr7P18880_001 and GSMUA_Achr7P18930_001). A C2H2-like zinc protein finger (GSMUA_Achr4P07200_001) is also potentially linked to Abscisic Acid and Gibberelin signaling. In addition, two genes were found to be orthologous to genes involved in gametophyte development, one on chromosome 3 (GSMUA_Achr3T22920_001) which is a putative Histidine Kinase CKI1 that, once muted, induced female sterility in *Arabidopsis* [51], and one on chromosome 4 (GSMUA_Achr4P07370_001) annotated as a leucine-rich repeat receptor-like serine/threonine-protein kinase that is orthologous to BAM1 and BAM2 in *Arabidopsis*, which were associated with male and female gametophyte functionality [52]. Interestingly, CKl1 was found in the region of chromosome 3 that exhibited the most significant association with seedlessness when analyzing the subset. Finally, two genes on chromosome 1 (GSMUA_Achr1P02650_001) and on chromosome 5 (GSMUA_Achr5P15620_001) may be involved in pollen tube development, while one additional gene on chromosome 3 (GSMUA_Achr3P01350_001) appeared orthologous to STAMENLESS 1 which is a zinc-finger protein involved in floral development in *Arabidopsi*s [53].

**Table 5:** List of candidate genes functionally annotated.

Chr	Gene name	Start	Stop	Description	Ortholog(s)^a^	Gene family ^b^	References ^c^	Pathways
**1**	GSMUA_Achr1P02650_001	2 169 774	2 170 710	Expansin-A1	At1g20190	GP000070	PMID:11641069	cell-wall-loosening, Pollen tube invasion
**3**	GSMUA_Achr3P01350_001	961 998	962 537	Zinc finger protein STAMENLESS 1-like	At1g13400	GP000363	PMID:19453444	Floral development
**3**	GSMUA_Achr3P22920_001	23 765 387	23 769 402	Histidine kinase CKI1	At2g47430	GP000157	PMID:20363773	Gametophyte development
**4**	GSMUA_Achr4P07190_001	5 358 776	5 360 344	Scarecrow-like protein 15	At4g36710	GP000046	PMID:21245327	Gibberellin signaling
**4**	GSMUA_Achr4P07200_001	5 364 860	5 367 289	C2H2-like zinc finger protein	-	GP005446	PMID:21571950	ABA signaling
								Gibberellin signaling
**4**	GSMUA_Achr4P07220_001	5 386 015	5 388 073	Indole-3-acetic acid-amido synthetase GH3.8	LOC_Os07g40290 (OsGH3.8)	GP015282	PMID:23136372	Auxin signaling
**4**	GSMUA_Achr4P07320_001	5 454 250	5 456 801	Scarecrow-like protein 4	At5g66770	GP000046	PMID:21245327	Gibberellin signaling
**4**	GSMUA_Achr4P07370_001	5 471 492	5 474 806	LRR receptor-like serine/threonine-protein kinase BAM1, BAM2	At5g65700 (BAM1), At3g49670 (BAM2)	GP069343	PMID:16367950	Male and female gametophyte
**5**	GSMUA_Achr5P15620_001	11 638 620	11 641 100	Pectate lyase 15	At4g13710	GP000253	PMID:1983191	Pollen tube growth development
**7**	GSMUA_Achr7P18880_001	21 725 684	21 732 717	Zeaxanthin epoxidase	LOC_Os04g37619 (ZEP)	GP018321	PMID:16482436	ABA signaling
**7**	GSMUA_Achr7P18930_001	21 748 033	21 748 452	AP2/ERF transcription factor	At3g16770 (RAP2-3)	GP015092	PMID:25253450	ABA signaling

Genes were identified through comparative genomics approaches (see methods).

^a^ Arabidopsis or rice gene with the highest homology to the Musa gene

^b^ Family ID from GreenPhyl data base (http://www.greenphyl.org/).

^c^ References supporting the potential of the candidate gene or gene family characterized in other plants.

## Discussion

### SNP dataset and Genetic Structure of the Panel

In this paper, we presented the selection, genotyping-by-sequencing and subsequent analyses of a panel of accessions deemed to be used in GWAS. The stringency of the parameters used to filter the data associated with the known presence of different genepools within the panel resulted in a reduced number of SNPs. It is known that the different subspecies of *M*. *acuminata* are heterogeneous, exhibiting various levels of diversity and heterozygosity [54] along with structural rearrangements [55]. It is therefore expected that the mapping of the reads on one of these subspecies, here *M*. *acuminata malaccensis*, would alter SNP calling for the others and for those of the cultivated accessions carrying genetic backgrounds different from *malaccensis*. Taking into account the expected highly hybrid status of most of the cultivated accessions, and the likely absence in the panel of at least one of the ancestral genepools [26], we intentionally allow few missing data in our dataset to avoid erroneous imputations.

Despite the careful selection of the genotypes to prevent possible repetitions due to clonality, the initial set of samples available did not preclude the presence of genetic structure among the panel. The analysis of the set of 5,544 SNPs performed with STRUCTURE confirmed the high levels of admixture of many cultivated accessions of bananas, including some originating from Papua, suggesting complex domestication schemes. Such schemes are in line with the hypothesis of multiple hybridization events following the circulation of forms of bananas pre-domesticated in multiple genepools as sources of the present cultivated banana diversity [3]. This pattern is also supported by the high levels of heterozygosity of the admixed cultivated varieties (0.35 in average). However and noticeably, a batch of cultivated accessions mostly originating from Papua is unadmixed and seems not to follow the accepted model. Cluster 2, the Papuan subset of the study, is indeed composed of those cultivated accessions and of individuals of the Papuan subspecies *M*. *acuminata banksii*. Therefore we may consider these cultivated accessions as directly domesticated from the local wild genepool. Interestingly, this subspecies of *M*. *acuminata* is believed to be the only of the subspecies predominantly selfing, while the others are supposed to be out-crossing. However, the levels of He observed in the unadmixed wild accessions in the different clusters identified by STRUCTURE, i.e. 0.15 in Cl. 1, 0.12 in Cl. 2 and 0.08 in Cl. 3, may argue for self-pollination in all three wild genepools. Nevertheless, we may consider the filtration performed on MAF > 5% partly responsible for this pattern. Wild hybrids logically exhibit higher He (0.32 in average).

Overall, the level of heterozygosity by individuals was lower in wild types than in edibles. The high levels of admixture observed in many of the cultivated accessions of the panel are likely mostly responsible for these levels of heterozygosity. The vegetative mode of propagation of the crop may also have impacted the heterozygosity observed in the cultivated accessions as it is admitted that the accumulation of mutations through clonal propagation promotes divergence between alleles for a specific locus (aka Meselson effect), resulting in an increase in heterozygosity [56], which would be consistent with our results.

### Pattern of LD

The pattern of LD, decreasing to half its initial value between 10 kb and 100 kb, then never reaching the level of background LD, is expected to limit the resolution of the association study. Therefore, the sizes of the exploration windows chosen for this study, 80 kb and higher in particular contexts, seem reasonable. On the other hand, the levels of structuring detected in many accessions of the panel, mostly unseeded but not only, must be taken into consideration. According to the results obtained from the study of the genetic structure, the panel is obviously composed of accessions with different genomic histories for both seeded and unseeded types. It is noticeable that the levels of heterozygosity of the unadmixed accessions of Cl. 1, Cl. 2 and Cl. 3 are very low and may reflect predominantly selfing populations, in which a slow decay of LD would be expected. On the contrary, the high levels of admixture detected in 59 accessions result from hybridization events between different genepools. These hybridizations should have broken LD to some extent, but may also have created long LD blocks, corresponding to the chromosome segments originating from the different parental populations of these admixed accessions. Indeed, structural heterozygosity in cultivated bananas is common, including in diploid accessions [57],[25],[55],[23],[58]. For those accessions that are concerned, we may consider that they are genomic mosaics, i.e. mosaics of discrete segments each with its own unique history. In such a context, the windows explored around the significantly associated SNPs may not be wide enough, as long-distance LD, corresponding to these segments, may occur. Unfortunately in this study, the small number of markers and the resulting low coverage of the genome by these markers do not allow a fine study of LD patterns.

### Genome-Wide Association Study for seedless phenotype

Given the LD pattern of the panel, the genome coverage of the selected SNPs is not optimal and we may have missed markers truly associated to our phenotype. In addition, and given the likely occurrence of long-distance LD, the possibility of detecting regions distantly associated with causal SNPs cannot be ignored even though we did not identify such cases. We aimed to present the panel and show its potential to successfully perform GWAS using easy-to-handle analysis tools, notably TASSEL, one of the most used software to perform association studies in crop plants. Given that the panel i) was suspected to exhibit complex and unbalanced levels of genomic structure [26], ii) is composed of a relatively small set of accessions, therefore decreasing imputation accuracy [59] and that iii) missing data imputation may introduce substantial biases in downstream genetics analyses [60], we intentionally filtered out missing data to avoid imputation and to get highly reliable markers. Further genotyping of this panel, using other techniques and other enzymes, should increase the coverage of the genome and allow a more precise detection of candidate genes.

Nevertheless, we identified 13 candidate regions potentially involved in the seedless phenotype in banana, among which six appeared also significantly associated when analysing the subset of 33 accessions. A previous GWAS performed on a carefully selected population composed of 95 individuals of *A*. *thaliana* successfully detected known loci linked to the different traits studied [61]. Therefore, we do believe in a true statistical association of most of these sites with the domestication phenotype based on i) the distributions of the p-values perfectly following the expected distribution under the assumption of significant associations (Fig 4C and 4G), ii) the stringent Bonferroni correction successfully applied to the SNPs detected and iii) the overall consistency of the results obtained between the different sets of accessions analysed (Table 4). We thus assume our candidate regions are strong candidates. The proportions of phenotypic variance explained by the associated SNPs were high, especially for the subset analysis (Table 4), but this pattern can be explained by different factors. Firstly, it could be due to the small sizes of the panel and of the subset. In addition, it could ensue from the qualitative nature of the phenotype studied here. Even though the seeded–unseeded phenotype corresponds to two different traits, i.e. parthenocarpy and female sterility, it may well be that both traits are driven by a small number of genes. Indeed, Simmonds [24] assumed one of them, parthenocarpy, to be driven by a major gene coupled with few minor ones. The banana reference genome exploration was performed on c.a. 80 kb windows around the regions being significantly associated with the trait studied. The 13 genomic regions explored yielded a total number of 176 genes. Through the comparative genomic approaches we used, we flagged eleven of them as being of particular interest in the context of seedlessness in banana, that is to say with possible links with parthenocarpy or female sterility. Finally, the panel is composed of accessions originating from different genepools and different geographic regions across South-East Asia and Near Oceania. Such sampling pattern increases the association power but can also induce genetic heterogeneity, i.e. different genes may underpin a same phenotype, and such pattern would weaken the correlation between the phenotype and a given variant [32].

### Candidate genes identified and pattern of domestication

The correction for both kinship and structure applied to the MLM succeeded in reducing the proportion of false-positive association (Fig 4C). The occurrence of a number of candidate regions is consistent with the known genetic basis of the seedless phenotype in banana. The genetic pathways involved in natural parthenocarpy are still unresolved but the increased levels of Auxin and GA observed in the fruits exhibiting naturally occurring parthenocarpy, including banana, [62],[12],[63] along with decreases in ABA [12], suggests that internal mechanisms mimic pollination to induce fruit set. In wild-type plants, fertilization and the subsequent formation of a seed induces an auxin signal that will directly allow the expression (or repression) of auxin-sensitive fruit initiation genes through, for example, the release of Auxin-Responsive Factors (ARF) (e.g. *Sl*ARF7 [64]). Auxin also induces a GA signal that will further allow the degradation of DELLA proteins and the subsequent activation of DELLA-repressed fruit initiation genes (reviewed in [65]). Other hormones such as ABA may also be involved but its role has not been characterized yet. Parthenocarpy in banana is believed to be caused by the combination of at least three genes among which one would be major and dominant [25]. Therefore, the identification of more than one candidate region was expected. Even though none of the six candidate genes potentially linked to the hormonal pathways of fruit set was strikingly obvious, we assume our results are consistent.

In addition of parthenocarpy, female sterility is also of importance in the seedless phenotype but its genetic determinism is not mandatory, as structural heterozygosity also induces sterility [25]. In this context, the analysis of the 33 accessions of the Papuan subset is of particular interest. Those accessions were indeed identified by STRUCTURE as unadmixed. The 27 cultivated accessions of this panel are the only cultivated accessions that are apparently not structural heterozygotes and for which a genetic determinism of female sterility is mandatory. Therefore, the identification of a gene orthologous to a gene inducing female sterility in *Arabidopsis* within the region exhibiting the lowest p-values for this analysis is striking. Indeed, in *Arabidopsis*, the loss-of-function of CKl1, for which the ortholog was found on the region of chromosome 3, induced the loss of female fertility [66],[67],[51]. No paralogs to this gene were found in the banana genome, suggesting that mutation involving banana CKl1 could have an impact on female fertility. In addition, the silencing of BAM1 and BAM2, for which an ortholog was found on chromosome 4, led to male sterility and to a drastic reduction of female fertility in *Arabidopsis* [52]. It was previously proposed that domestication of banana occurred in several centres of South-East Asia and independently in Papua [26]. In such context, it may well be that different prevalent mechanisms are underlying the traits of interests that were selected by the first farmers in each region. It also supports past authors’ assumptions that female sterility in banana could also be due to genetic factors [10].

Finally, the association studies were performed on a general phenotype that we named seedlessness. Seedlessness, induced by parthenocarpy combined with sterility, is the main trait associated with the domestication syndrome in banana. However, it is more than likely that domestication resulted in the concomitant selection of other traits, such as for example the propensity for vegetative propagation, which may have been unintentionally considered in this association study. The resolution of the genomic basis of banana domestication will thus require further investigation, including both genomics and phenomics approaches. However, we may have performed here a substantial first step in that direction. In addition, the absence of seeds in edible bananas and the need to incorporate new traits in the cultivated genepools result in the wide use of wild accessions as female parents in the crosses performed by breeders. This practice results usually in various levels of seed production within the progeny obtained. If validated, the potential role of CKl1 in female sterility would be of major importance for marker-assisted selection.

### Feasibility, Promises and Future of Genome-Wide Association Studies in Banana

The main goal of this study was to present a set of banana accessions allowing performing GWAS on this vegetatively propagated, weakly fertile, potentially hybrid and polyploid crop species. We used this panel to perform a GWAS on a qualitative trait as a proof of concept for feasibility in banana. The set of accessions presented here appeared successful in detecting, through GWAS, interesting candidate genes for a phenotype that is of prime importance for banana breeding, i.e. the capacity to set unseeded fruits without the need of prior pollination. We assume thus that GWAS in banana is not only a feasible but also a promising approach, at least for traits underpinned by few loci, conditional to stringency in the various steps of the association process.

The selection of a small number of accessions for this GWAS panel was largely due to the relatively small number of different diploid *M*. *acuminata* clones available, but not only. A number of constraints weigh on banana phenotyping, which is far more difficult to perform than in cereals. Even though early mass screening for disease resistance are being developed under greenhouse conditions [68], banana traits linked to yield or organoleptic properties can hardly be measured in greenhouses. Field-testing approaches for perennial crops are highly demanding in space and time. For example, a banana field experiment requires 6 m² per plant, 12 to 18 months for the plants to mature and evaluation should be performed over several crop cycles. Under that scope, the small size of the panel undoubtedly will ease further phenotyping and evaluation. This panel of accessions is freely available for distribution at the ITC and documented on MGIS (http://www.crop-diversity.org/mgis/content/gwas-panel-gbs) with links to all SNP datasets that are publically available and visualizable. Even though GWAS for complex traits underlined by genes with moderate or small effects has not been tested yet with this panel, it is likely that these elements will allow a broader use of GWAS in banana and will speed-up the process of discovering genes coding for traits of interest at the level of the banana community [69].

Finally, Brachi et al [70] proposed different schemes to further improve the accuracy and resolution of the results of GWAS in plants. However, these recommendations mostly affected the elaboration of the plant samples to be used. It was proposed to develop multiple bi-parental crosses or multi-parent intercrosses for the study of broadly adaptive traits and then to identify and sample regional subsamples to study locally adaptive traits. Such strategies are uneasy, if not impossible, to achieve in banana, due to the difficulty in performing sexual crosses on partially or fully sterile accessions; the space and time necessary to maintain, characterize and evaluate extensive field collections for that crop; and finally to the relatively low number of diploid cultivated accessions existing worldwide. In this context, the present paper not only opens perspectives for banana genomics and molecular breeding, it also shows that a limited number of accessions properly selected and the use of simple tools available can facilitate GWAS in complex crops.

## Supporting Information

S1 Fig a.Frequency histogram of the pairwise genetic distances calculated following the IAM for 224 diploid accessions with pure *M*. *acuminata* genetic background genotyped with 498 DArT markers. b. NJ tree based on the dissimilarity matrix calculated following Sokal and Michener [71] index for 224 diploid accessions with pure *M*. *acuminata* genetic background genotyped with 498 DArT markers.
(PDF)

S2 Fig SNP density by chromosome on a sliding window of 50kb. Graph were generated using the SNP density tool provided by SNIplay (http://sniplay.cirad.fr) [72].
(PDF)

S3 Fig Quantile-Quantile and Manhattan plots obtained performing GWAS on the panel and on the Papuan subset with MAF > 5%. (PDF)

S1 Table Barcode adpaters used for the Genotyping-By-Sequencing. (PDF)

S2 Table List of analyzed genes on chromosomes 1, 3, 4, 7 and 9. Classification of the gene families was performed using seq2families provided by GreenPhyl (http://www.greenphyl.org/cgi-bin/seq2families.cgi) [44].
(XLSX)

## References

[1] Available: http://faostat3.fao.org/home/E. In: FAOstat.

[2] De Langhe E, Langhe ED, Vrydaghs L, Maret P de, Perrier X, Denham T. Why Bananas Matter: An introduction to the history of banana domestication. Ethnobot Res Appl. 2009;7: 165–177. 10.17348/era.7.0.165-177

[3] Perrier X, De Langhe E, Donohue M, Lentfer C, Vrydaghs L, Bakry F, et al Multidisciplinary perspectives on banana (Musa spp.) domestication. Proc Natl Acad Sci. 2011; 10.1073/pnas.1102001108 PMC3136277 21730145

[4] Huang X, Wei X, Sang T, Zhao Q, Feng Q, Zhao Y, et al Genome-wide association studies of 14 agronomic traits in rice landraces. Nat Genet. 2010;42: 961–967. 10.1038/ng.695 20972439

[5] Pasam RK, Sharma R, Malosetti M, van Eeuwijk FA, Haseneyer G, Kilian B, et al Genome-wide association studies for agronomical traits in a world wide spring barley collection. BMC Plant Biol. 2012;12: 16 10.1186/1471-2229-12-16 22284310 PMC3349577

[6] Morris GP, Rhodes DH, Brenton Z, Ramu P, Thayil VM, Deshpande S, et al Dissecting genome-wide association signals for loss-of-function phenotypes in sorghum flavonoid pigmentation traits. G3 Bethesda Md. 2013;3: 2085–2094. 10.1534/g3.113.008417 PMC3815067 24048646

[7] Zila CT, Samayoa LF, Santiago R, Butrón A, Holland JB. A genome-wide association study reveals genes associated with fusarium ear rot resistance in a maize core diversity panel. G3 Bethesda Md. 2013;3: 2095–2104. 10.1534/g3.113.007328 PMC3815068 24048647

[8] Courtois B, Audebert A, Dardou A, Roques S, Ghneim-Herrera T, Droc G, et al Genome-wide association mapping of root traits in a japonica rice panel. PloS One. 2013;8: e78037 10.1371/journal.pone.0078037 24223758 PMC3818351

[9] Tian F, Bradbury PJ, Brown PJ, Hung H, Sun Q, Flint-Garcia S, et al Genome-wide association study of leaf architecture in the maize nested association mapping population. Nat Genet. 2011;43: 159–162. 10.1038/ng.746 21217756

[10] Simmonds NW. The evolution of the bananas. London (GBR): Longmans; 1962.

[11] Ozga JA, Huizen R van, Reinecke DM. Hormone and Seed-Specific Regulation of Pea Fruit Growth. Plant Physiol. 2002;128: 1379–1389. 10.1104/pp.010800 11950986 PMC154265

[12] Talon M, Zacarias L, Primo-Millo E. Hormonal changes associated with fruit set and development in mandarins differing in their parthenocarpic ability. Physiol Plant. 1990;79: 400–406. 10.1111/j.1399-3054.1990.tb06759.x

[13] Fos M, Nuez F, García-Martínez JL. The gene pat-2, which induces natural parthenocarpy, alters the gibberellin content in unpollinated tomato ovaries. Plant Physiol. 2000;122: 471–480. 10677440 10.1104/pp.122.2.471 PMC58884

[14] Ding J, Chen B, Xia X, Mao W, Shi K, Zhou Y, et al Cytokinin-induced parthenocarpic fruit development in tomato is partly dependent on enhanced gibberellin and auxin biosynthesis. PloS One. 2013;8: e70080 10.1371/journal.pone.0070080 23922914 PMC3726760

[15] Menezes CB de, Maluf WR, Azevedo SM de, Faria MV, Nascimento IR, Nogueira DW, et al Inheritance of parthenocarpy in summer squash (Cucurbita pepo L.). Genet Mol Res GMR. 2005;4: 39–46. 15841434

[16] Kim IS, Okubo H, Fujieda K. Endogenous levels of IAA in relation to parthenocarpy in cucumber (Cucumis sativus L.). Sci Hortic. 1992;52: 1–8. 10.1016/0304-4238(92)90002-T

[17] Tiwari A, Vivian-Smith A, Voorrips RE, Habets MEJ, Xue LB, Offringa R, et al Parthenocarpic potential in Capsicum annuum L. is enhanced by carpelloid structures and controlled by a single recessive gene. BMC Plant Biol. 2011;11: 143 10.1186/1471-2229-11-143 22018057 PMC3214887

[18] Miyatake K, Saito T, Negoro S, Yamaguchi H, Nunome T, Ohyama A, et al Development of selective markers linked to a major QTL for parthenocarpy in eggplant (Solanum melongena L.). TAG Theor Appl Genet Theor Angew Genet. 2012;124: 1403–1413. 10.1007/s00122-012-1796-8 22301906

[19] Beraldi D, Picarella ME, Soressi GP, Mazzucato A. Fine mapping of the parthenocarpic fruit (pat) mutation in tomato. TAG Theor Appl Genet Theor Angew Genet. 2004;108: 209–216. 10.1007/s00122-003-1442-6 14564391

[20] Pascual L, Blanca JM, Cañizares J, Nuez F. Transcriptomic analysis of tomato carpel development reveals alterations in ethylene and gibberellin synthesis during pat3/pat4 parthenocarpic fruit set. BMC Plant Biol. 2009;9: 67 10.1186/1471-2229-9-67 19480705 PMC2700107

[21] Gorguet B, Eggink PM, Ocaña J, Tiwari A, Schipper D, Finkers R, et al Mapping and characterization of novel parthenocarpy QTLs in tomato. Theor Appl Genet. 2008;116: 755–767. 10.1007/s00122-007-0708-9 18231773 PMC2271080

[22] Simmonds NW. The Development of the Banana Fruit. J Exp Bot. 1953;4: 87–105. 10.1093/jxb/4.1.87

[23] Adeleke MTV, Pillay M, Okoli BE. Relationships between Meiotic Irregularities and Fertility in Diploid and Triploid *Musa* L. Cytologia (Tokyo). 2004;69: 387–393. 10.1508/cytologia.69.387

[24] Simmonds NW. The Development of the Banana Fruit. J Exp Bot. 1953;4: 87–105. 10.1093/jxb/4.1.87

[25] Dodds KS, Simmonds NW. Sterility and parthenocarpy in diploid hybrids of musa. Heredity. 1948;2: 101–117. 10.1038/hdy.1948.6 18863987

[26] Sardos J, Perrier X, Dolezel J, Hribova E, Christelova P, Kilian A, et al. DArT whole genome profiling provides insights on the evolution and taxonomy of edible Banana (*Musa* spp.). Submitted. 10.1093/aob/mcw170 PMC5155597 27590334

[27] Elshire RJ, Glaubitz JC, Sun Q, Poland JA, Kawamoto K, Buckler ES, et al A Robust, Simple Genotyping-by-Sequencing (GBS) Approach for High Diversity Species. PLoS ONE. 2011;6: e19379 10.1371/journal.pone.0019379 21573248 PMC3087801

[28] Meirmans PG, Van Tienderen PH. genotype and genodive: two programs for the analysis of genetic diversity of asexual organisms. Mol Ecol Notes. 2004;4: 792–794. 10.1111/j.1471-8286.2004.00770.x

[29] Douhovnikoff V, Dodd RS. Intra-clonal variation and a similarity threshold for identification of clones: application to Salix exigua using AFLP molecular markers. Theor Appl Genet. 2003;106: 1307–1315. 10.1007/s00122-003-1200-9 12748783

[30] Perrier X, Flori A, Bonnot F. Data analysis methods In: Hamon P., Seguin M., Perrier X., Glaszmann J. C. Ed., Genetic diversity of cultivated tropical plants. Enfield, Science Publishers Montpellier; 2003 pp. 43–76.

[31] Perrier X, Jacquemoud-Collet, JP. **DARwin** software. CIRAD. 2006. Available: http://darwin.cirad.fr/

[32] Korte A, Farlow A. The advantages and limitations of trait analysis with GWAS: a review. Plant Methods. 2013;9: 29 10.1186/1746-4811-9-29 23876160 PMC3750305

[33] Glaubitz JC, Casstevens TM, Lu F, Harriman J, Elshire RJ, Sun Q, et al TASSEL-GBS: A High Capacity Genotyping by Sequencing Analysis Pipeline. PLoS ONE. 2014;9: e90346 10.1371/journal.pone.0090346 24587335 PMC3938676

[34] D’Hont A, Denoeud F, Aury J-M, Baurens F-C, Carreel F, Garsmeur O, et al The banana (Musa acuminata) genome and the evolution of monocotyledonous plants. Nature. 2012; 10.1038/nature11241 22801500

[35] Droc G, Lariviere D, Guignon V, Yahiaoui N, This D, Garsmeur O, et al The Banana Genome Hub. Database. 2013;2013: bat035–bat035. 10.1093/database/bat035 23707967 PMC3662865

[36] Li H, Durbin R. Fast and accurate short read alignment with Burrows–Wheeler transform. Bioinformatics. 2009;25: 1754–1760. 10.1093/bioinformatics/btp324 19451168 PMC2705234

[37] Weir BS, Cockerham CC. Estimating F-Statistics for the Analysis of Population Structure. Evolution. 1984;38: 1358–1370. 10.2307/2408641 28563791

[38] Belkhir K, Borsa P, Chikhi L, Raufaste N, Bonhomme F. GENETIX 4.05, logiciel sous Windows TM pour la génétique des populations. Lab Génome Popul Interact CNRS UMR. 1996;5000: 1996–2004.

[39] Pritchard JK, Stephens M, Donnelly P. Inference of population structure using multilocus genotype data. Genetics. 2000;155: 945–959. 10835412 10.1093/genetics/155.2.945 PMC1461096

[40] Falush D, Stephens M, Pritchard JK. Inference of Population Structure Using Multilocus Genotype Data: Linked Loci and Correlated Allele Frequencies. Genetics. 2003;164: 1567–1587. 12930761 10.1093/genetics/164.4.1567 PMC1462648

[41] Saitou N, Nei M. The neighbor-joining method: a new method for reconstructing phylogenetic trees. Mol Biol Evol. 1987;4: 406–425. 3447015 10.1093/oxfordjournals.molbev.a040454

[42] Barrett JC, Fry B, Maller J, Daly MJ. Haploview: analysis and visualization of LD and haplotype maps. Bioinformatics. 2005;21: 263–265. 10.1093/bioinformatics/bth457 15297300

[43] Sun L, Craiu RV, Paterson AD, Bull SB. Stratified false discovery control for large-scale hypothesis testing with application to genome-wide association studies. Genet Epidemiol. 2006;30: 519–530. 10.1002/gepi.20164 16800000

[44] Rouard M, Guignon V, Aluome C, Laporte M-A, Droc G, Walde C, et al GreenPhylDB v2.0: comparative and functional genomics in plants. Nucleic Acids Res. 2011;39: D1095–1102. 10.1093/nar/gkq811 20864446 PMC3013755

[45] Cenci A, Guignon V, Roux N, Rouard M. Genomic analysis of NAC transcription factors in banana (Musa acuminata) and definition of NAC orthologous groups for monocots and dicots. Plant Mol Biol. 2014; 10.1007/s11103-013-0169-2 PMC4151281 24570169

[46] Katoh K, Kuma K, Toh H, Miyata T. MAFFT version 5: improvement in accuracy of multiple sequence alignment. Nucl Acids Res. 2005;33: 511–518. 10.1093/nar/gki198 15661851 PMC548345

[47] Talavera G, Castresana J. Improvement of phylogenies after removing divergent and ambiguously aligned blocks from protein sequence alignments. Syst Biol. 2007;56: 564–577. 10.1080/10635150701472164 17654362

[48] Guindon S, Gascuel O. A simple, fast, and accurate algorithm to estimate large phylogenies by maximum likelihood. Syst Biol. 2003;52: 696–704. 14530136 10.1080/10635150390235520

[49] Anisimova M, Gascuel O. Approximate likelihood-ratio test for branches: A fast, accurate, and powerful alternative. Syst Biol. 2006;55: 539–552. 10.1080/10635150600755453 16785212

[50] Staswick PE, Serban B, Rowe M, Tiryaki I, Maldonado MT, Maldonado MC, et al Characterization of an Arabidopsis enzyme family that conjugates amino acids to indole-3-acetic acid. Plant Cell. 2005;17: 616–627. 10.1105/tpc.104.026690 15659623 PMC548830

[51] Deng Y, Dong H, Mu J, Ren B, Zheng B, Ji Z, et al Arabidopsis histidine kinase CKI1 acts upstream of histidine phosphotransfer proteins to regulate female gametophyte development and vegetative growth. Plant Cell. 2010;22: 1232–1248. 10.1105/tpc.108.065128 20363773 PMC2879746

[52] DeYoung BJ, Bickle KL, Schrage KJ, Muskett P, Patel K, Clark SE. The CLAVATA1-related BAM1, BAM2 and BAM3 receptor kinase-like proteins are required for meristem function in Arabidopsis. Plant J Cell Mol Biol. 2006;45: 1–16. 10.1111/j.1365-313X.2005.02592.x 16367950

[53] Xiao H, Tang J, Li Y, Wang W, Li X, Jin L, et al STAMENLESS 1, encoding a single C2H2 zinc finger protein, regulates floral organ identity in rice. Plant J. 2009;59: 789–801. 10.1111/j.1365-313X.2009.03913.x 19453444

[54] Carreel F, Fauré S, De León DG, Lagoda P, Perrier X, Bakry F, et al Evaluation de la diversité génétique chez les bananiers diploïdes (Musa sp). Genet Sel Evol. 1994;26: 125s–136s.

[55] Shepherd K. Cytogenetics of the genus Musa. IPGRI; 1999.

[56] Balloux F, Lehmann L, de Meeûs T. The population genetics of clonal and partially clonal diploids. Genetics. 2003;164: 1635–1644. 12930767 10.1093/genetics/164.4.1635 PMC1462666

[57] Dodds K. Genetical and cytological studies of Musa. V. Certain edible diploids. J Genet. 10.1007/BF02986785 21010998

[58] Hippolyte I, Jenny C, Gardes L, Bakry F, Rivallan R, Pomies V, et al Foundation characteristics of edible Musa triploids revealed from allelic distribution of SSR markers. Ann Bot. 2012; 10.1093/aob/mcs010 PMC3310492 22323428

[59] Browning SR, Browning BL. Rapid and Accurate Haplotype Phasing and Missing-Data Inference for Whole-Genome Association Studies By Use of Localized Haplotype Clustering. Am J Hum Genet. 2007;81: 1084–1097. 10.1086/521987 17924348 PMC2265661

[60] Fu Y-B. Genetic diversity analysis of highly incomplete SNP genotype data with imputations: an empirical assessment. G3 Bethesda Md. 2014;4: 891–900. 10.1534/g3.114.010942 PMC4025488 24626289

[61] Aranzana MJ, Kim S, Zhao K, Bakker E, Horton M, Jakob K, et al Genome-Wide Association Mapping in Arabidopsis Identifies Previously Known Flowering Time and Pathogen Resistance Genes. PLoS Genet. 2005;1: e60 10.1371/journal.pgen.0010060 16292355 PMC1283159

[62] Khalifah RA. Gibberellin-like Substances from the Developing Banana Fruit. Plant Physiol. 1966;41: 771–773. 16656319 10.1104/pp.41.5.771 PMC1086422

[63] Talon M, Zacarias L, Primo-Millo E. Gibberellins and parthenocarpic ability in developing ovaries of seedless mandarins. Plant Physiol. 1992;99: 1575–1581. 16669076 10.1104/pp.99.4.1575 PMC1080666

[64] de Jong M, Wolters-Arts M, García-Martínez JL, Mariani C, Vriezen WH. The Solanum lycopersicum AUXIN RESPONSE FACTOR 7 (SlARF7) mediates cross-talk between auxin and gibberellin signalling during tomato fruit set and development. J Exp Bot. 2011;62: 617–626. 10.1093/jxb/erq293 20937732 PMC3003806

[65] Sundberg E, Østergaard L. Distinct and Dynamic Auxin Activities During Reproductive Development. Cold Spring Harb Perspect Biol. 2009;1: a001628 10.1101/cshperspect.a001628 20457563 PMC2882118

[66] Pischke MS, Jones LG, Otsuga D, Fernandez DE, Drews GN, Sussman MR. An Arabidopsis histidine kinase is essential for megagametogenesis. Proc Natl Acad Sci U S A. 2002;99: 15800–15805. 10.1073/pnas.232580499 12426401 PMC137796

[67] Hejátko J, Pernisová M, Eneva T, Palme K, Brzobohatý B. The putative sensor histidine kinase CKI1 is involved in female gametophyte development in Arabidopsis. Mol Genet Genomics MGG. 2003;269: 443–453. 10.1007/s00438-003-0858-7 12774227

[68] Dita MA, Waalwijk C, Paiva LV, Jr.M.T S, Kema GHJ. A greenhouse bioassay for the Fusarium oxysporum f. sp. cubense x Grand naine” (Musa, AAA, Cavendish subgroup) interaction. 2011; Available: https://cgspace.cgiar.org/handle/10568/42421

[69] Available: http://www.musanet.org/ [Internet].

[70] Brachi B, Morris GP, Borevitz JO. Genome-wide association studies in plants: the missing heritability is in the field. Genome Biol. 2011;12: 232 10.1186/gb-2011-12-10-232 22035733 PMC3333769

[71] Sokal R, Michener C. A statistical method for evaluating systematic relationships. iniv. kansas sci. bull., 38: 1409–1438. Prim Product Ecol Factors Lake Maggiore. 1958;127.

[72] Dereeper A, Nicolas S, Le Cunff L, Bacilieri R, Doligez A, Peros J-P, et al SNiPlay: a web-based tool for detection, management and analysis of SNPs. Application to grapevine diversity projects. BMC Bioinformatics. 2011;12: 134 10.1186/1471-2105-12-134 21545712 PMC3102043

