# Supplementary figures and images for: A Genome-Wide Association Study on the Seedless Phenotype in Banana (Musa spp.) Reveals the Potential of a Selected Panel to Detect Candidate Genes in a Vegetatively Propagated Crop
Source: PLoS One. 2016 May 4;11(5):e0154448. doi: 10.1371/journal.pone.0154448 (PMC4856271; doi:10.1371/journal.pone.0154448)

a.

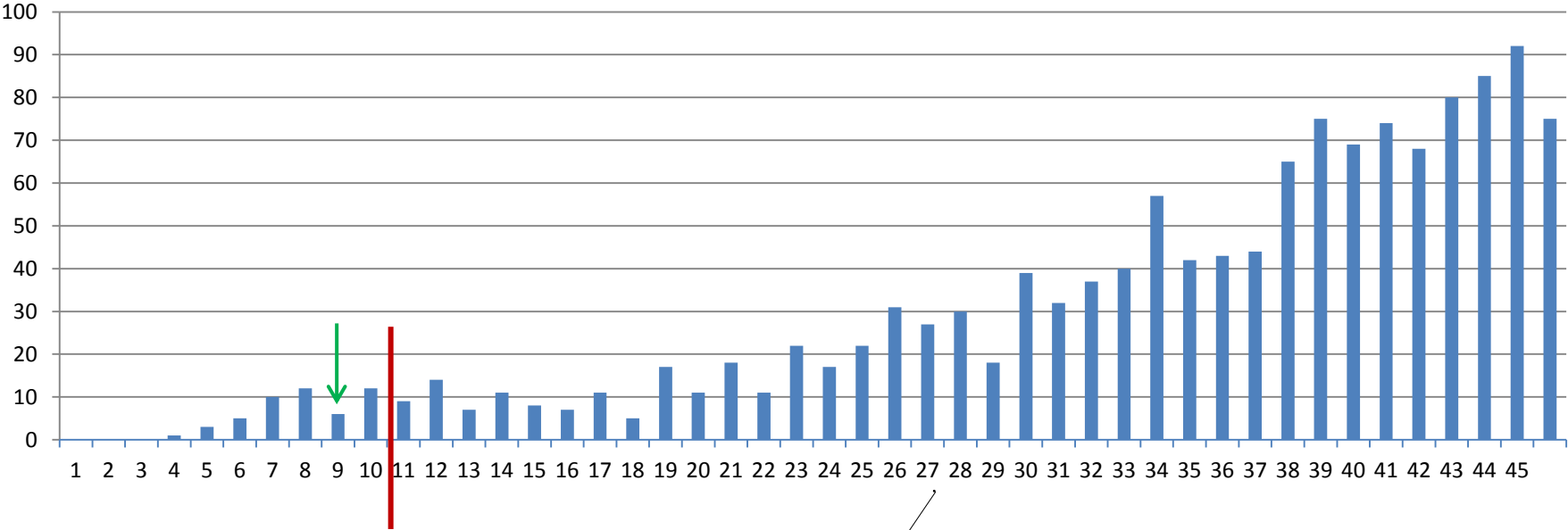

b.

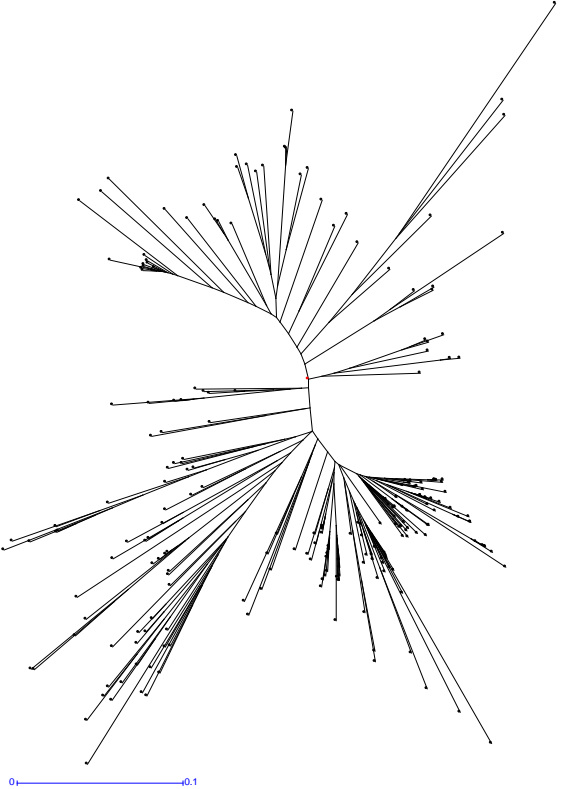

Supplement: S1 Fig — a.Frequency histogram of the pairwise genetic distances calculated following the IAM for 224 diploid accessions with pure M. acuminata genetic background genotyped with 498 DArT markers. b. NJ tree based on the dissimilarity matrix calculated following Sokal and Michener [71] index for 224 diploid accessions with pure M. acuminata genetic background genotyped with 498 DArT markers. (PDF) [file pone.0154448.s001.pdf]

Panel MAF 5% GLM

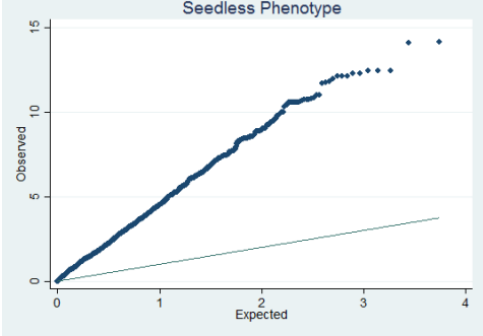

Panel MAF 5% MLM K + Q (K=4)

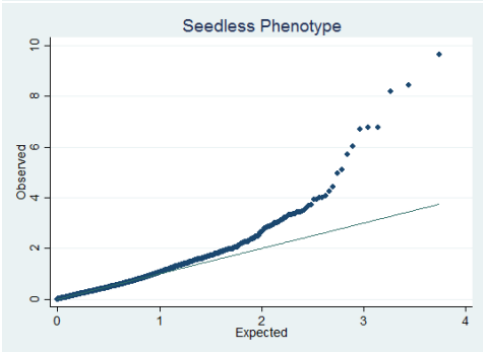

Subset MAF 5% GLM

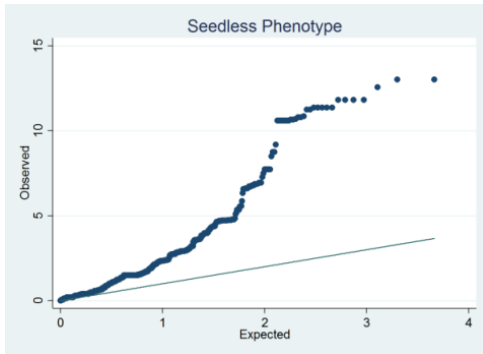

Subset MAF 5% MLM K

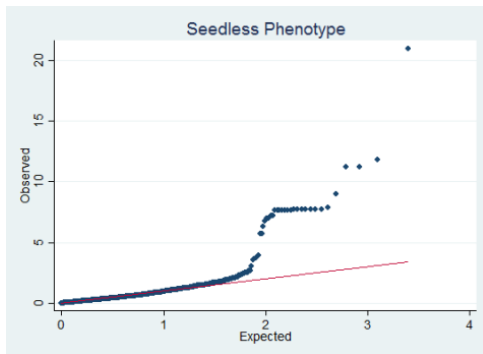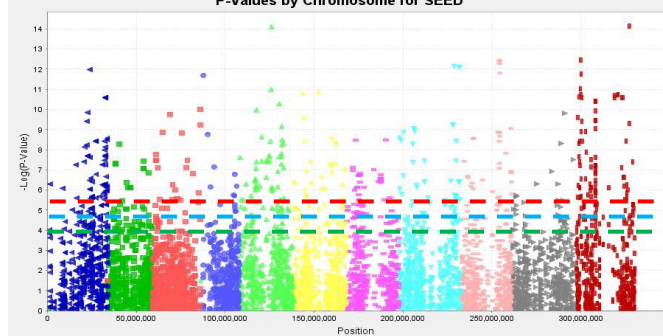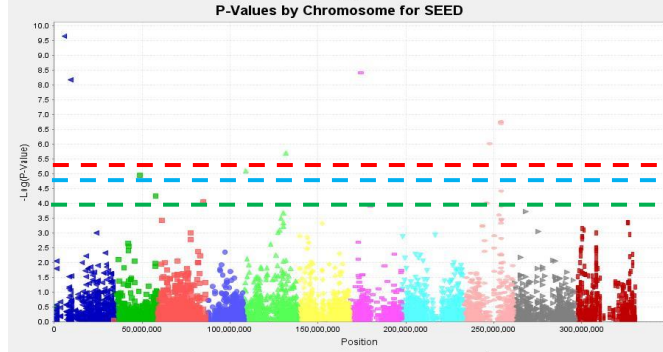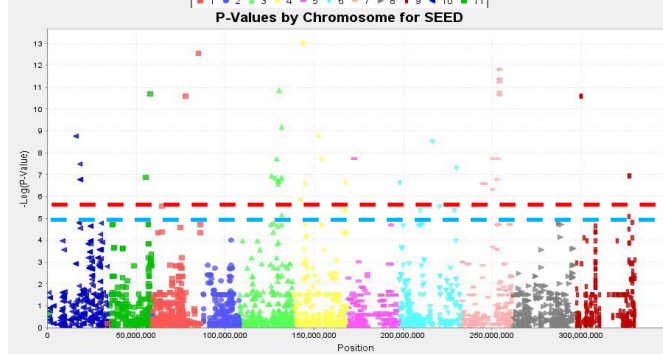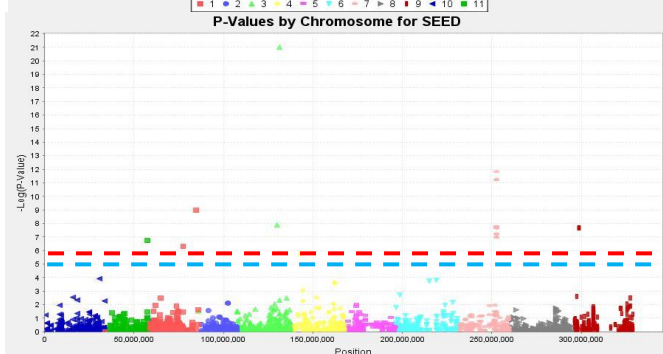

Supplement: S3 Fig — (PDF) [file pone.0154448.s003.pdf]
