# Supplementary material for: A Genome-Wide Association Study on the Seedless Phenotype in Banana (Musa spp.) Reveals the Potential of a Selected Panel to Detect Candidate Genes in a Vegetatively Propagated Crop
Source: PLoS One. 2016 May 4;11(5):e0154448. doi: 10.1371/journal.pone.0154448 (PMC4856271; doi:10.1371/journal.pone.0154448)
Supplement: S2 Fig — Graph were generated using the SNP density tool provided by SNIplay (http://sniplay.cirad.fr) [72]. (PDF) [file pone.0154448.s002.pdf]

S3 figure: SNP density by chromosome on a sliding window of 50kb. Graph were generated using the SNP density tool provided by SNIplay (http://sniplay.cirad.fr) [85]

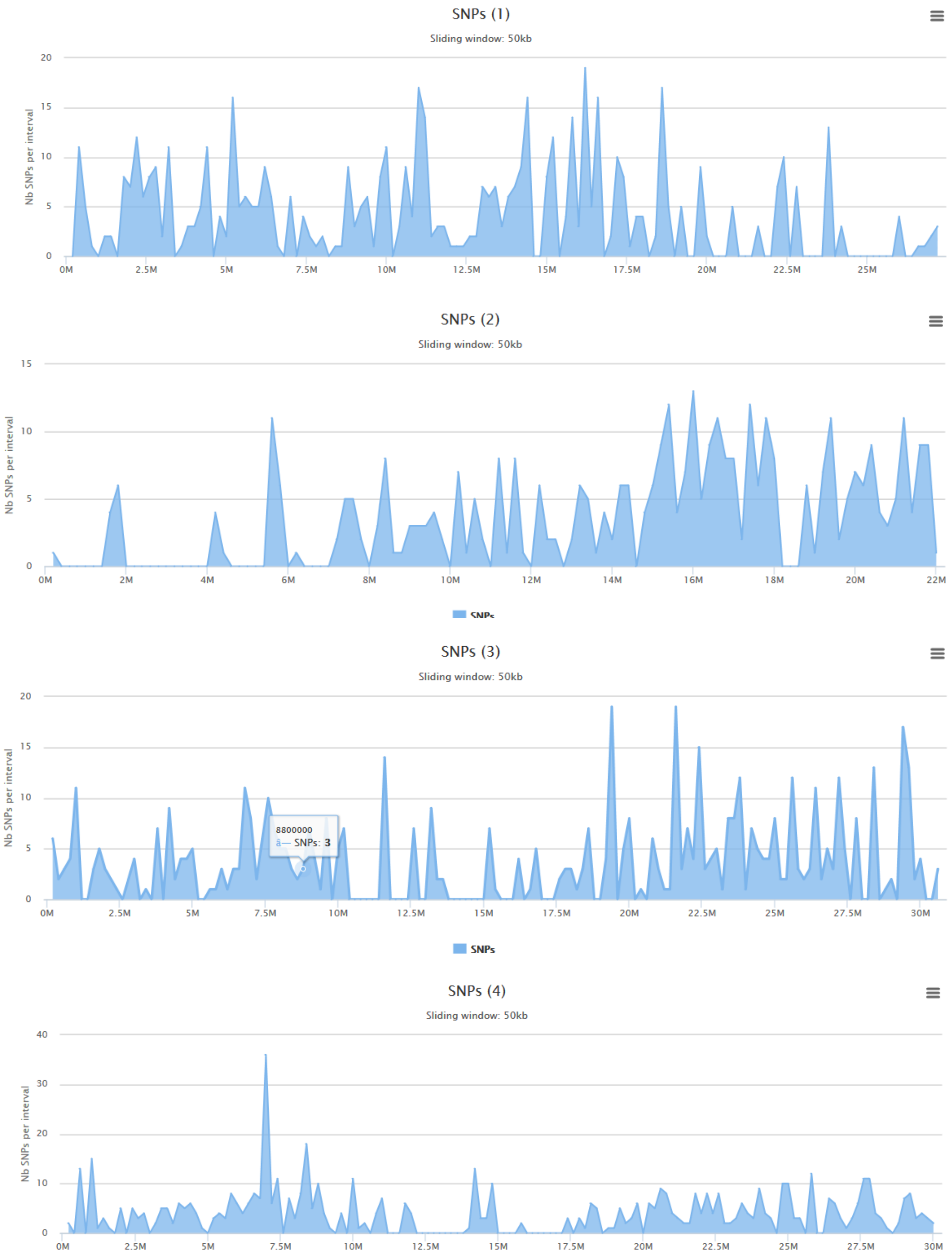

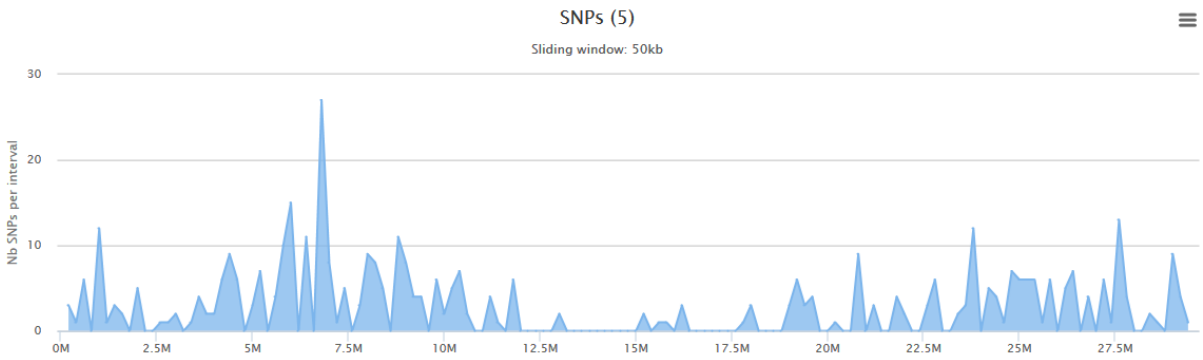
SNPs (5)
Sliding window: 50kb
Nb SNPs per interval
30
20
10
0
0M
2.5M
5M
7.5M
10M
12.5M
15M
17.5M
20M
22.5M
25M
27.5M

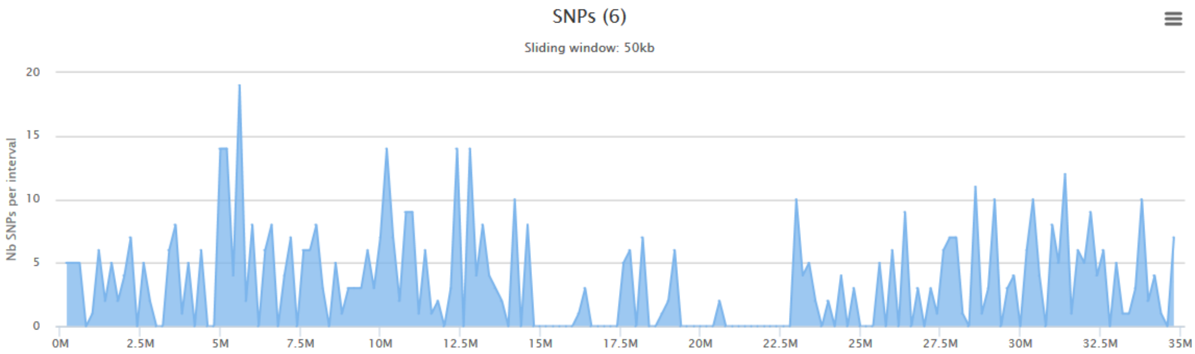
SNPs (6)
Sliding window: 50kb
Nb SNPs per interval
20
15
10
5
0
0M
2.5M
5M
7.5M
10M
12.5M
15M
17.5M
20M
22.5M
25M
27.5M
30M
32.5M
35M

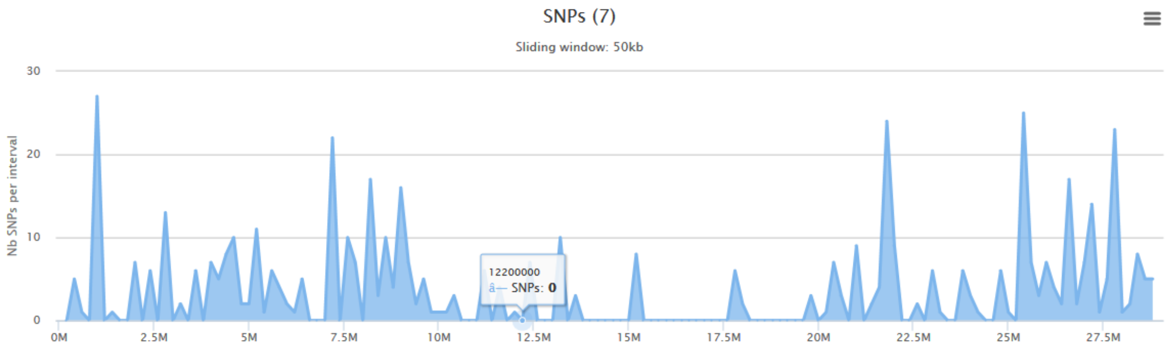
SNPs (7)
Sliding window: 50kb
Nb SNPs per interval
30
20
10
0
12200000
â— SNPs: 0
0M
2.5M
5M
7.5M
10M
12.5M
15M
17.5M
20M
22.5M
25M
27.5M

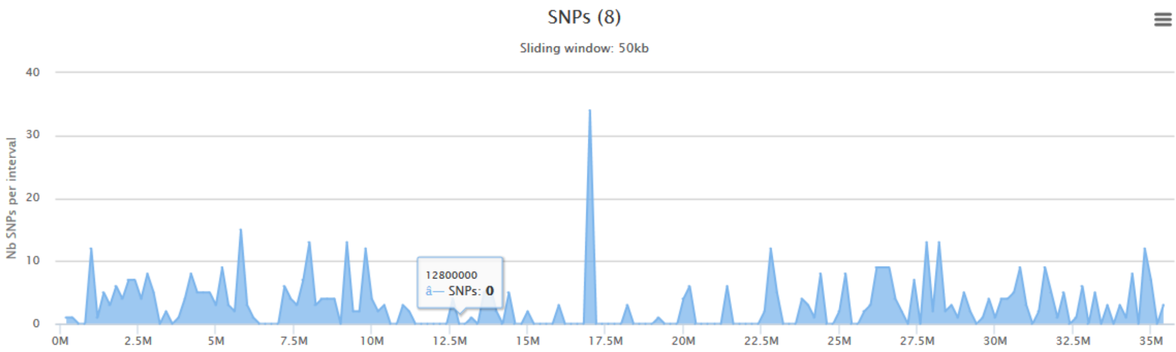
SNPs (8)
Sliding window: 50kb
Nb SNPs per interval
40
30
20
10
0
12800000
â— SNPs: 0
0M
2.5M
5M
7.5M
10M
12.5M
15M
17.5M
20M
22.5M
25M
27.5M
30M
32.5M
35M

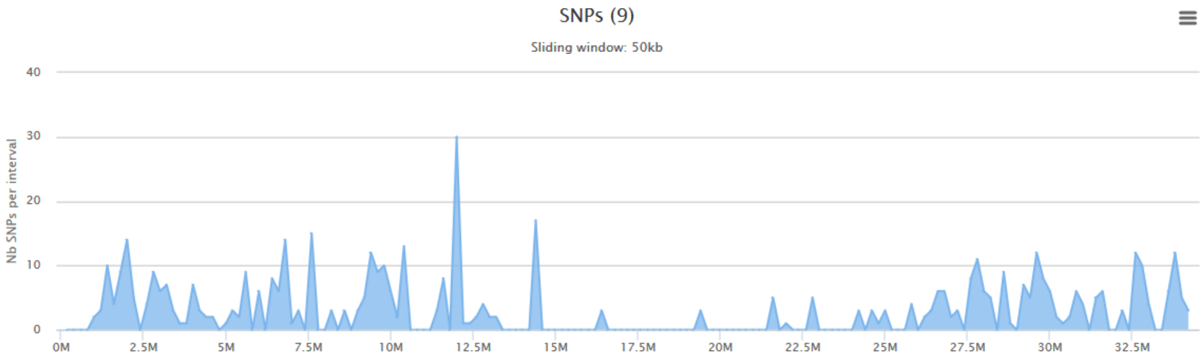
SNPs (9)
Sliding window: 50kb
Nb SNPs per interval
40
30
20
10
0
0M
2.5M
5M
7.5M
10M
12.5M
15M
17.5M
20M
22.5M
25M
27.5M
30M
32.5M

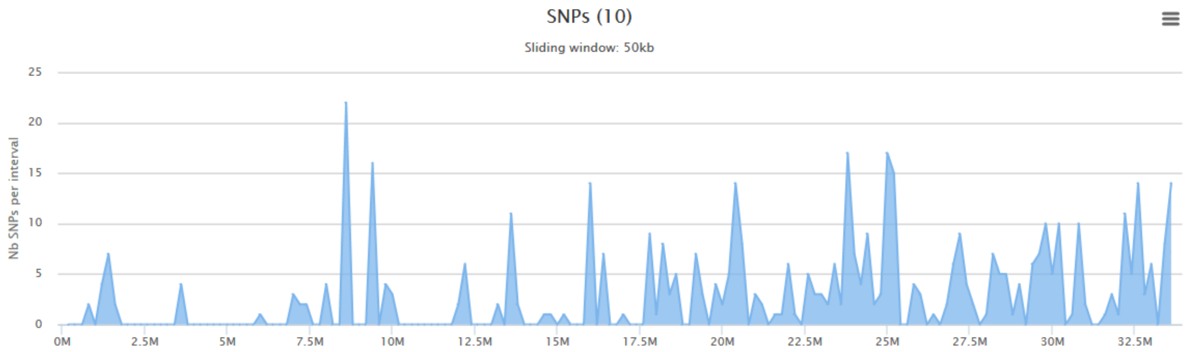
SNPs (10)
Sliding window: 50kb
Nb SNPs per interval
25
20
15
10
5
0
0M
2.5M
5M
7.5M
10M
12.5M
15M
17.5M
20M
22.5M
25M
27.5M
30M
32.5M

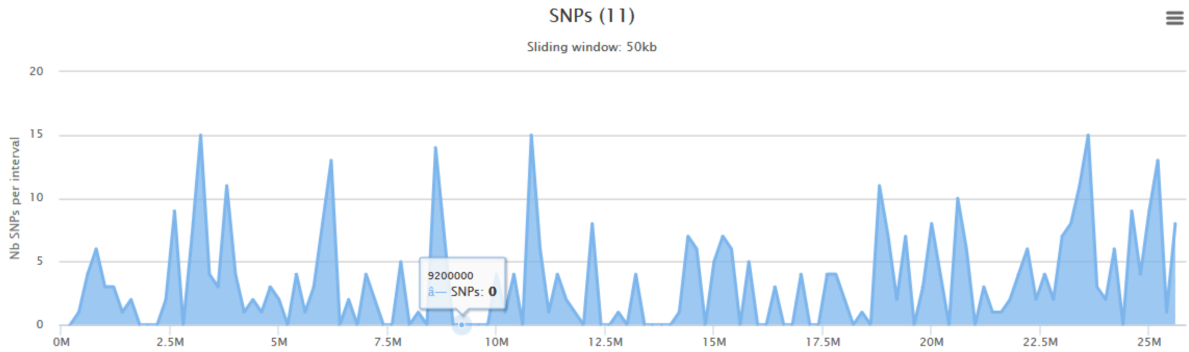
SNPs (11)
Sliding window: 50kb
Nb SNPs per interval
20
15
10
5
0
9200000
SNPs: 0
0M
2.5M
5M
7.5M
10M
12.5M
15M
17.5M
20M
22.5M
25M
