# Supplementary material for: A Genome-Wide Association Study on the Seedless Phenotype in Banana (Musa spp.) Reveals the Potential of a Selected Panel to Detect Candidate Genes in a Vegetatively Propagated Crop
Source: PLoS One. 2016 May 4;11(5):e0154448. doi: 10.1371/journal.pone.0154448 (PMC4856271; doi:10.1371/journal.pone.0154448)
Supplement: S1 Table — (PDF) [file pone.0154448.s004.pdf]

S2 Table: Barcode adpaters used for the Genotyping-By-Sequencing.

| | Top strand 5’ – 3’ | Bottom strand 5’ – 3’ |
|---|---|---|
| Barcode adapter | ACACTCTTTCCCTACACGACGCTCTTCCGATCT[barcode]TGCA | [barcode]AGATCGGAAGAGCGTCGTGTAGGGAAAGAGTGT |
| Common adapter | AGATCGGAAGAGCGGTTCAGCAGGAATGCCGAG | CTCGGCATTCCTGCTGAACCGCTCTTCCGATCTTGCA |
